# Methods for Detecting Early Warnings of Critical Transitions in Time Series Illustrated Using Simulated Ecological Data

**DOI:** 10.1371/journal.pone.0041010

**Published:** 2012-07-17

**Authors:** Vasilis Dakos, Stephen R. Carpenter, William A. Brock, Aaron M. Ellison, Vishwesha Guttal, Anthony R. Ives, Sonia Kéfi, Valerie Livina, David A. Seekell, Egbert H. van Nes, Marten Scheffer

**Affiliations:** 1 Department of Aquatic Ecology and Water Quality Management, Wageningen University, Wageningen, The Netherlands; 2 Integrative Ecology Group, Estación Biológica de Doñana, Sevilla, Spain; 3 Center for Limnology, University of Wisconsin, Madison, Wisconsin, United States of America; 4 Department of Economics, University of Wisconsin, Madison, Wisconsin, United States of America; 5 Harvard Forest, Harvard University, Petersham, Massachusetts, United States of America; 6 Centre for Ecological Sciences, Indian Institute of Science, Bangalore, India; 7 Department of Zoology, University of Wisconsin, Madison, Wisconsin, United States of America; 8 Institut des Sciences de l’Evolution, CNRS, Université de Montpellier II, Montpellier, France; 9 School of Environmental Sciences, University of East Anglia, Norwich, United Kingdom; 10 Department of Environmental Sciences, University of Virginia, Charlottesville, Virginia, United States of America; Rensselaer Polytechnic Institute, United States of America

## Abstract

Many dynamical systems, including lakes, organisms, ocean circulation patterns, or financial markets, are now thought to have tipping points where critical transitions to a contrasting state can happen. Because critical transitions can occur unexpectedly and are difficult to manage, there is a need for methods that can be used to identify when a critical transition is approaching. Recent theory shows that we can identify the proximity of a system to a critical transition using a variety of so-called ‘early warning signals’, and successful empirical examples suggest a potential for practical applicability. However, while the range of proposed methods for predicting critical transitions is rapidly expanding, opinions on their practical use differ widely, and there is no comparative study that tests the limitations of the different methods to identify approaching critical transitions using time-series data. Here, we summarize a range of currently available early warning methods and apply them to two simulated time series that are typical of systems undergoing a critical transition. In addition to a methodological guide, our work offers a practical toolbox that may be used in a wide range of fields to help detect early warning signals of critical transitions in time series data.

## Introduction

The Earth’s past has been characterized by rapid and often unexpected punctuated shifts in temperature and climatic conditions [1], lakes and coral reefs have shifted among alternative states [2], neural cells move regularly between different dynamical regimes [3], and financial markets are notorious for abrupt shifts. The gradual change in some underlying condition (or *driver*), such as the accumulation of phosphorus in a lake or the increasing flux of freshwater from melting ice sheets into the ocean, can bring a system closer to a catastrophic bifurcation point (a ‘tipping point’) causing a loss of resilience in the sense that even small perturbations can invoke a shift to an alternative state [2], [4]. In most cases, however, information about the drivers or the values at which systemic responses are so easily triggered (*critical thresholds*) is difficult to acquire (but see [5]). Nonetheless, these sudden transition incur large costs as restoration to the previous conditions is difficult or sometimes even impossible [2].

To overcome these challenges, numerous studies have suggested the use of generic early warning signals (or *leading indicators*) that can detect the proximity of a system to a tipping point [6]. Such indicators are based on common mathematical properties of phenomena that appear in a broad range of systems as they approach a catastrophic bifurcation [6]. An important application of these leading indicators is their potential real-time use as warnings of increased risk for upcoming transitions. However, they also may be used to rank instances of a system (e.g. different patients, individual coral reefs, different markets etc.) according to their proximity to a critical threshold.

**Table 1:** Early warning signals for critical transitions.

		Phenomenon			
	Method/Indicator	Rising memory	Rising variability	Flickering	Ref.
**metrics**	Autocorrelation at-lag-1	x			[23]
	Autoregressive coefficient of AR(1) model	x			[19]
	Return rate (inverse of AR(1) coefficient)	x			[23]
	Detrended fluctuation analysis indicator	x			[7]
	Spectral density	x			[20]
	Spectral ratio (of low to high frequencies)	x			[25]
	Spectral exponent	x			[this paper]
	Standard deviation		x	x	[28]
	Coefficient of variation		x	x	[28]
	Skewness		x	x	[29]
	Kurtosis		x	x	[25]
	Conditional heteroskedasticity		x	x	[32]
	BDS test		x	x	[10]
**models**	Time-varying AR(p) models	x	x		[38]
	Nonparametric drift-diffusion-jump models	x	x	x	[16]
	Threshold AR(p) models			x	[38]
	Potential analysis (potential wells estimator)			x	[43]

Leading indicator or method, the primary underlying dynamical phenomenon associated with it, and the original reference in which it was developed.

Several empirical studies have now demonstrated that leading indicators can be found in a variety of systems. Increases in autocorrelation has been documented prior to past climatic transitions [7], [8], increased variability has been shown before extinction in zooplankton lab experiments [9], and before an experimentally induced regime shift in a lake food web [10], whereas decreases in recovery rates have been demonstrated in chemical reactions [11], lasers [12], or in the plankton [13]. However, the statistical detection of leading indicators in both past events and in real time remains challenging for at least two reasons. First, there is a lack of appropriate data. High frequency sampling and designed experimentation have been proposed as potential solutions that can improve the detection of leading indicators [6], [10]. In many important cases, however, high frequency sampling or experiments are impossible. Furthermore, in many systems, sampling schemes are designed explicitly to avoid temporal autocorrelation, which is, in fact, needed for the accurate application and assessment of leading indicators (see worked examples below).

**Figure 1:**
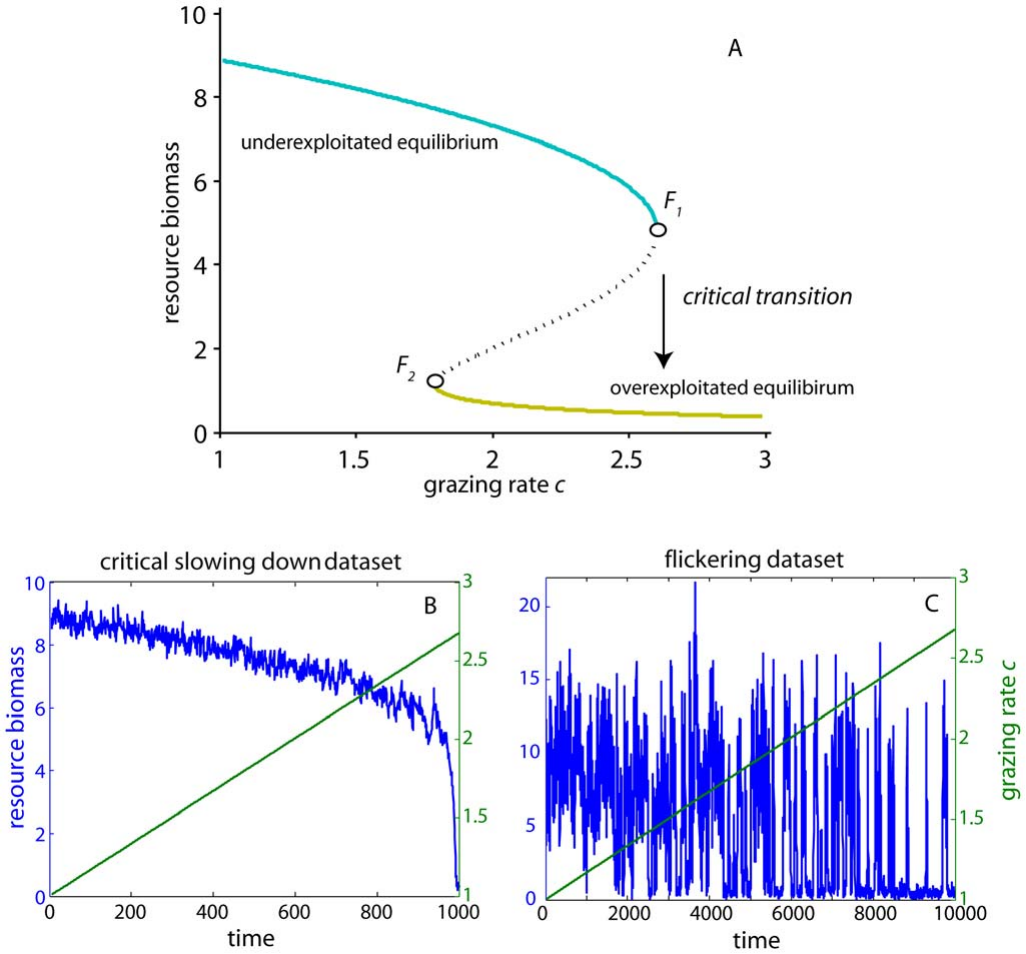
Two simulated paths towards a critical transition to overexploitation that resulted in the critical slowing down and flickering datasets used in the study. (A) Bifurcation diagram of an ecological model of a logistically growing resource under harvesting. As grazing rate *c* increases (*x* axis), resource biomass gradually declines up to a critical grazing threshold that the resource undergoes a critical transition through a fold bifurcation (*F_1_*). At this bifurcation the resource collapses to the alternative overexploited state. If grazing rate *c* is restored, resource biomass returns to the previous underexploited state at another threshold (*F_2_*). [solid lines represent equilibria, dashed line marks the boundary between the two basins of attraction between the underexploited (cyan) and overexploited (yellow) states] (B) Critical slowing down simulated dataset of resource biomass (blue line) for gradually increasing grazing rate (green line). (C) Flickering simulated dataset of resource biomass (blue line) for gradually increasing grazing rate (green line).

Second, there is no clear framework for the application and detection of leading indicators. Different approaches have emerged in different fields [14] and have been applied to different types of transitions [15]. For instance, most leading indicators are based on detecting changes in the stability properties of a system around its equilibrium under a weak stochastic regime [6], whereas alternative approaches have been developed for systems experiencing highly noisy regimes [16]. As the literature is rapidly expanding, there is an urgent need for a coherent methodological framework and a comparison between approaches.

**Figure 2:**
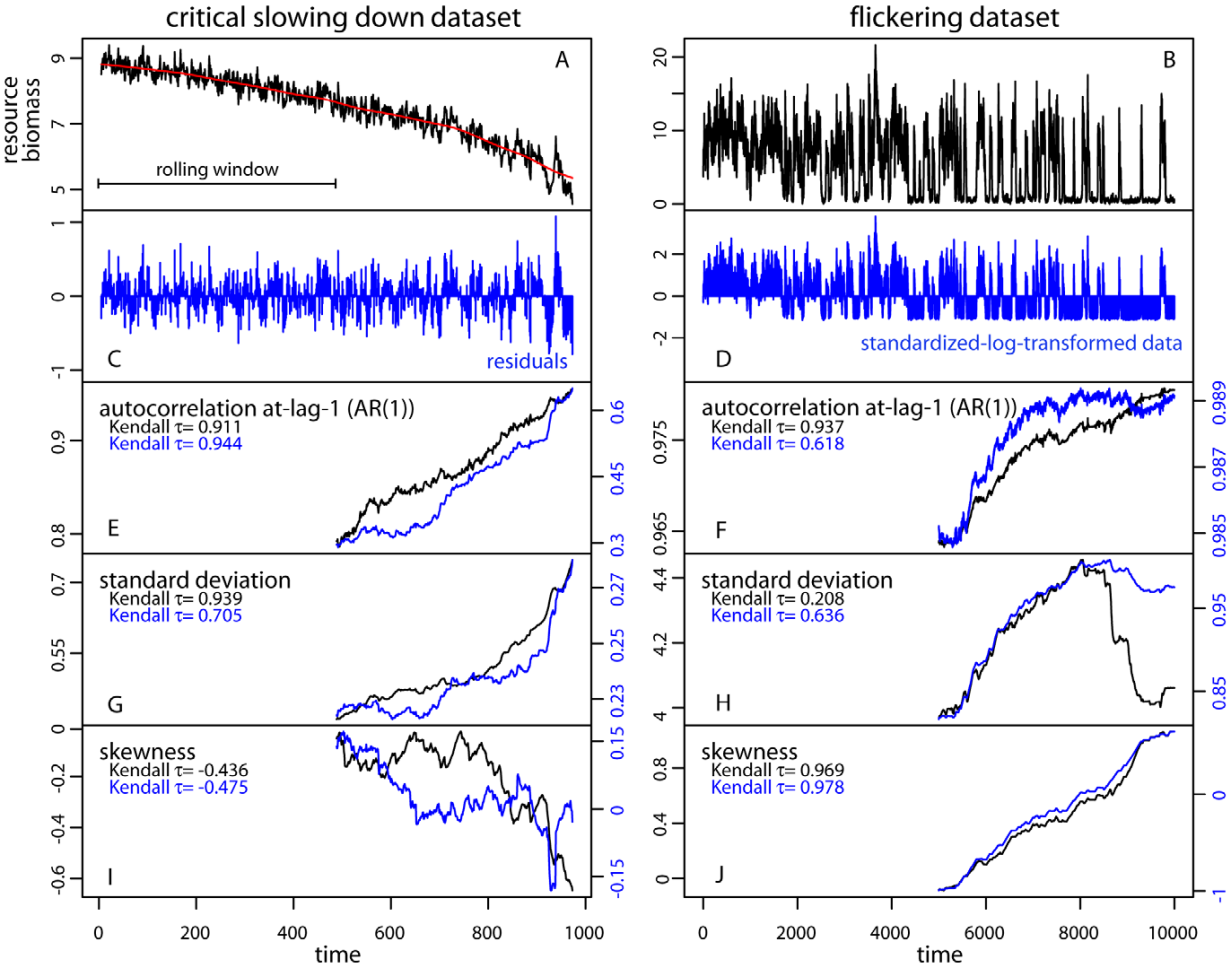
Metric-based rolling window indicators estimated on the critical slowing down and flickering datasets. (A, B) Time series of the state variable. (C) Residual time series after applying Gaussian filtering. (D) Standardized time series after log-transforming the flickering dataset. (E–I) Autocorrelation at-lag-1 (AR1), standard deviation, and skewness estimated within rolling windows of half the size of either the original, filtered or transformed time series. The Kendall τ indicate the strength of the trend in the indicators along the time series. [red line is the Gaussian filtering; black lines correspond to the metrics estimated on the original data, blue lines correspond to the metrics estimated on the residual or transformed data].

Here we present a methodological guide for using leading indicators for detecting critical transitions in time series. For this, we apply available leading indicators to two example datasets generated from a simple ecological model that is known to undergo a critical transition to an alternative state. While most of these methods have been applied to real-world data in papers that we cite, such applications inevitably depend on specific details (e.g. missing values, data transformation, coping with too-long sampling intervals or too-short time series) that make it difficult to compare the methods themselves. The exact location and nature of the critical transition is also ambiguous for real-world data. Therefore we gather issues of data preprocessing in a separate section (see “Step 1. Preprocessing” below), and illustrate the methods using simulated data with known, clearly defined critical transitions. The structure of the paper is as follows. First, we describe two categories of leading indicators: *metric-based* and *model-based* indicators. Second, we present the ecological model we use to generate the time series we use to detect critical transitions. Third, we show how each indicator is applied to the two simulated time series. We provide computer code alongside the worked-out examples. Last, we review the sensitivity and limitations of each indicator and discuss their interpretation. We trust that the framework and the tools we provide will encourage testing the ability of these indicators to detect upcoming transitions in real systems.

**Figure 3:**
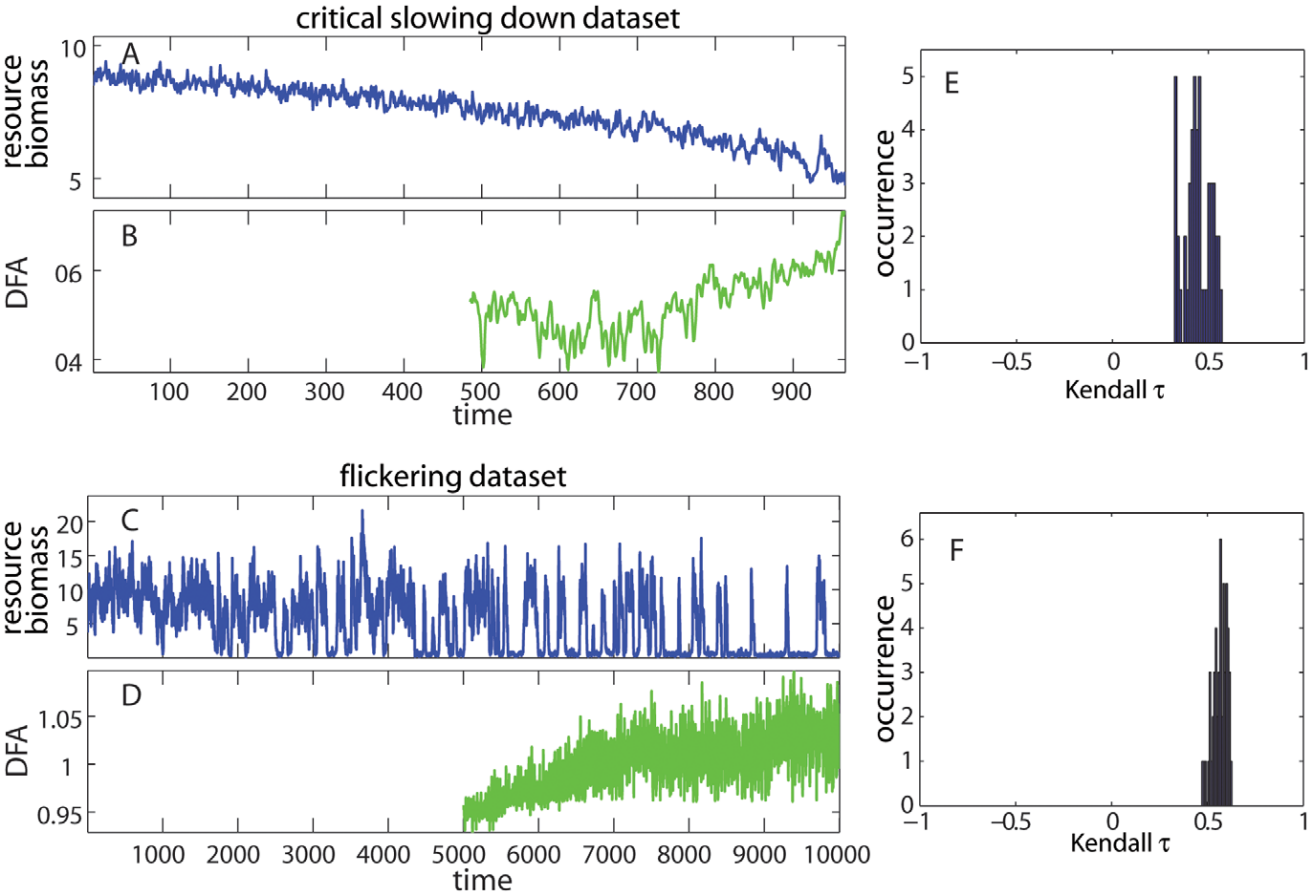
Detrended fluctuation analysis exponents (DFA) estimated on the critical slowing down and flickering datasets. (A, C) Time series of the state variable. (B, D). DFA estimated within rolling windows of half the size of the original time series applied after linear detrending. (E, F) Distributions of Kendall τ rank correlations indicate a positive trend in the indicators along the time series for different sizes of rolling windows.

## Methods

We group leading indicators of critical transitions into two broad categories*: metric-based* and *model-based* indicators (Table 1). Both types of indicators reflect changes in the properties of the observed time series of a system that is generated by a general process:
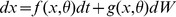
(1)where *x* is the state of the system, *f(x,θ)* describes the deterministic part of the system, and *g(x,θ)dW* determines how stochasticity interacts with the state variable; *dW* is a white noise process. A slow change in the underlying conditions (drivers), *θ*, moves the system close to a threshold where a transition may occur. *Metric-based* indicators quantify changes in the statistical properties of the time series generated by equation 1 without attempting to fit the data with a specific model structure. *Model-based* methods quantify changes in the time series by attempting to fit the data to a model that is based on the general structure of equation 1. The ultimate goal of both types of indicators is to capture changes in the ‘memory’ (i.e. correlation structure) and variability of a time series and to determine if they follow patterns as predicted by models of critical transitions, while the system is approaching a transition into an alternative dynamic regime (Table 1).

**Figure 4:**
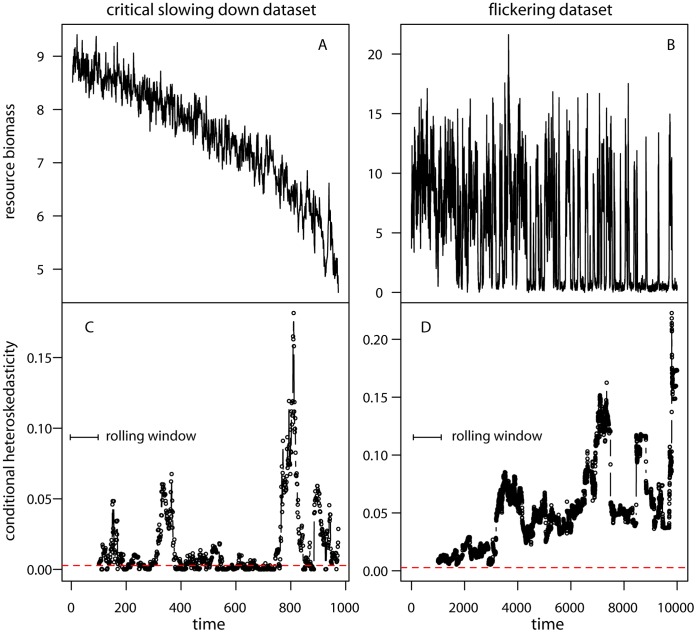
Conditional heteroskedasticity estimated on the critical slowing down and flickering datasets. (A, B) Time series of the state variable. (C, D) CH estimated within rolling windows of 10% the size of the original time series. CH was applied to the residuals of the best fit AR(p) on the original datasets. Values of CH above the dashed red line are significant (*P* = 0.1).

### Metric-based Indicators

#### Autocorrelation and spectral properties

The rate of return to equilibrium following a (small) perturbation slows down as systems approach critical transitions [17]. This slow return rate has been termed “critical slowing down” [18] and can be detected by changes in the correlation structure of a time series. In particular, critical slowing down causes an increase in the ‘short-term memory’ ( = correlation at low lags) of a system prior to a transition [19], [20].

**Table 2:** BDS statistic estimated on the critical slowing down and flickering datasets with measurement error.

BDS statistic		First-difference detrending			AR(1) residuals			GARCH(0,1) residuals		
		ε (standard deviation)								
		0.5	0.75	1	0.5	0.75	1	0.5	0.75	1
***critical slowing down dataset***										
embedding dimension	2	9.434*	9.013*	8.424*	9.499*	8.911*	8.462*	6.748*	6.343*	5.605*
	3	8.346*	8.042*	7.497*	8.379*	7.639*	7.307*	6.089*	5.469*	4.802*
***flickering dataset***										
embedding dimension	2	16.033*	16.33*	16.754*	15.476*	15.866*	16.332*	1.087	0.974	0.820
	3	17.599*	17.821*	18.039*	16.999*	17.304*	17.577*	3.472**	3.389**	3.155**

* *P*<0.001,

** *P* = 0.001.

In all cases, the BDS test was significantly identifying nonlinearity after 1,000 bootstrapping iterations, except for GARCH residuals from the flickering dataset.

Autocorrelation is the simplest way to measure slowing down: an increase in *autocorrelation at-lag-1* indicates that the state of the system has become increasingly similar between consecutive observations [19]. There are at least three alternative ways to measure autocorrelation at-lag-1. The most straightforward is to estimate the first value of the autocorrelation function, 
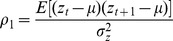
, where *μ* is the mean and *σ* the variance of variable *z_t_* [21]. Alternatively one can use a conditional least-squares method to fit an autoregressive model of order 1 (linear AR(1)-process) of the form; *z_t_*_+1_ = *α_1_z_t_* + *ε_t_*, where *ε_t_* is a Gaussian white noise process, and *α_1_* is the autoregressive coefficient [21]. *ρ_1_* and *α_1_* are mathematically equivalent [21]. Slowing down can also be expressed as *return rate*: the inverse of the first-order term of a fitted autoregressive AR(1) model [1/*α_1_*] [22], [23]. The *return rate* has also been expressed as [1-*α_1_*], which reflects the proportion of the distance from equilibrium that decays away at each time step [10].

**Figure 5:**
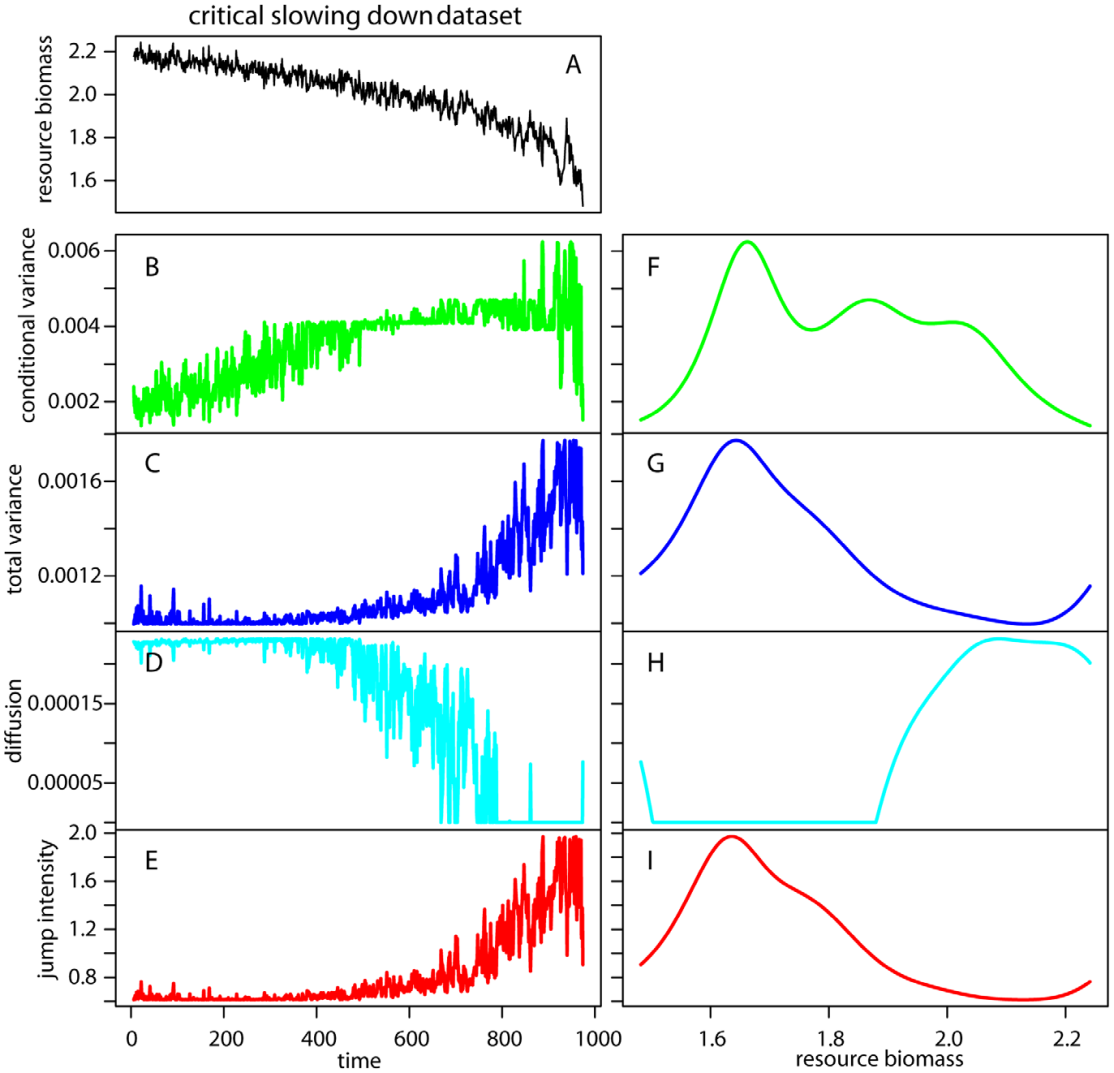
Nonparametric drift-diffusion-jump metrics in the critical slowing down dataset. (A) Time series of the state variable (resource biomass). (B, F) Conditional variance versus time and resource biomass respectively. (C, G) Total variance versus time and resource biomass respectively. (D, H) Diffusion versus time and resource biomass respectively. (G, I) Jump intensity versus time and resource biomass respectively.

Whereas autocorrelation at-lag-1 ignores changes in correlation structure at higher lags, *power spectrum analysis* can reveal changes in the complete spectral properties of a time series prior to a transition. Power spectrum analysis partitions the amount of variation in a time series into different frequencies [21]. A system close to a transition tends to show spectral reddening: higher variation at low frequencies [20]. Changes in the power spectra of a time series also can be expressed in different ways: by estimating the entire power spectrum and observing a shift in the power of *spectral densities* to lower frequencies [20]; by estimating the *spectral exponent* of the spectral density based on the slope of a linear fitted model on a double-log scale of spectral density *versus* frequency [24]; or by estimating the *spectral ratio* of the spectral density at low frequency (e.g. 0.05) to the spectral density at high frequency (e.g. 0.5) [25].

**Figure 6:**
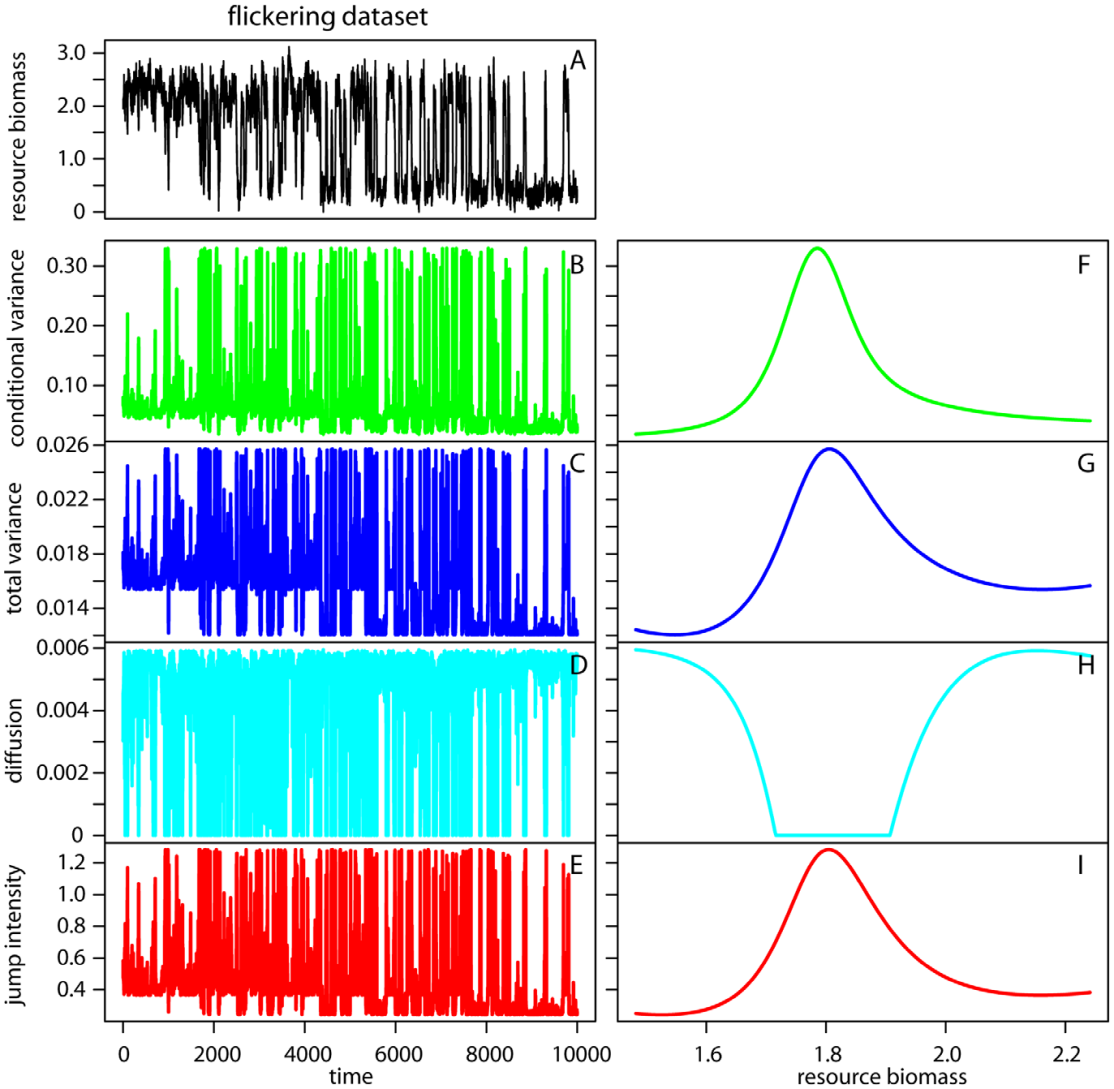
Nonparametric drift-diffusion-jump metrics in the flickering dataset. (A) Time series of the state variable (resource biomass). (B, F) Conditional variance versus time and biomass respectively. (C, G) Total variance versus time and resource biomass respectively. (D, H) Diffusion versus time and resource biomass respectively. (G, I) Jump intensity versus time and resource biomass respectively.

#### Detrended fluctuation analysis

Detrended fluctuation analysis (DFA) can be used to measure increases in short- and mid-term ‘memory’ in a time series of a system close to transition. Instead of estimating correlations at a given lag (like autocorrelation at-lag-1), DFA estimates a range of correlations by extracting the fluctuation function of a time series of size *s*. If the time series is long-term power-law correlated, the fluctuation function F(s) increases as a power law; 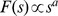, where *a* is the DFA fluctuation exponent [26]. The DFA fluctuation exponent is then rescaled to give a DFA indicator, which is usually estimated in time ranges between 10 and 100 time units, and which reaches value 1 (rescaled from 1.5) at a critical transition [7]. Although, the DFA captures similar information as autocorrelation at-lag-1, it is more data demanding (it requires >100 points for robust estimation) [27].

**Figure 7:**
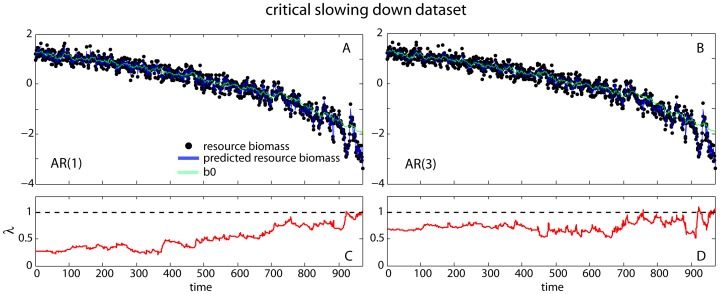
Fitting time-varying AR(n) models to the critical slowing down and flickering datasets. (A) Time-varying AR(1) model fit to the critical slowing down dataset. Differences between the fitted trajectory (blue line) and the simulated data (black dots) are attributed to measurement error. The green line gives the time-varying estimate of *b*_0_(*t*) from the AR(1). Parameter estimates are: *b*_0_ = 1.263, *b*_1_ = 0.278, *σ*_ε_ = 0.154, *σ*_α_ = 0.113, and *σ*_1_ = 0.015, and the log likelihood is 150.838. (B) Time-varying AR(3) model fit to the critical slowing down dataset. Parameter estimates are: *b*_0_ = 1.284, *b*_1_ = 0.342, *b*_2_ = 0.02, *b*_3_ = 0.139, *σ*_ε_ = 0.116, *σ*_α_ = 0.141, *σ*_1_ = 0.019, *σ*_2_ =  0.015, and *σ*_3_<0.001, and the log likelihood is 154.102. (C, D) The inverse of the characteristic root for the AR(1) and AR(3) time-varying models respectively.

#### Variance

Slow return rates back to a stable state close to a transition also can make the system state drift widely around the stable state. Moreover, strong disturbances potentially can push the system across boundaries of alternative states – a phenomenon termed *flickering*. Both slowing down and flickering will cause *variance* to increase prior to a complete transition [6]. Variance is the second moment around the mean *μ* of a distribution and serves as early warning measured either as *standard deviation*: 
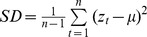
 or alternatively as the *coefficient of variation* 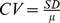 [28].

**Figure 8:**
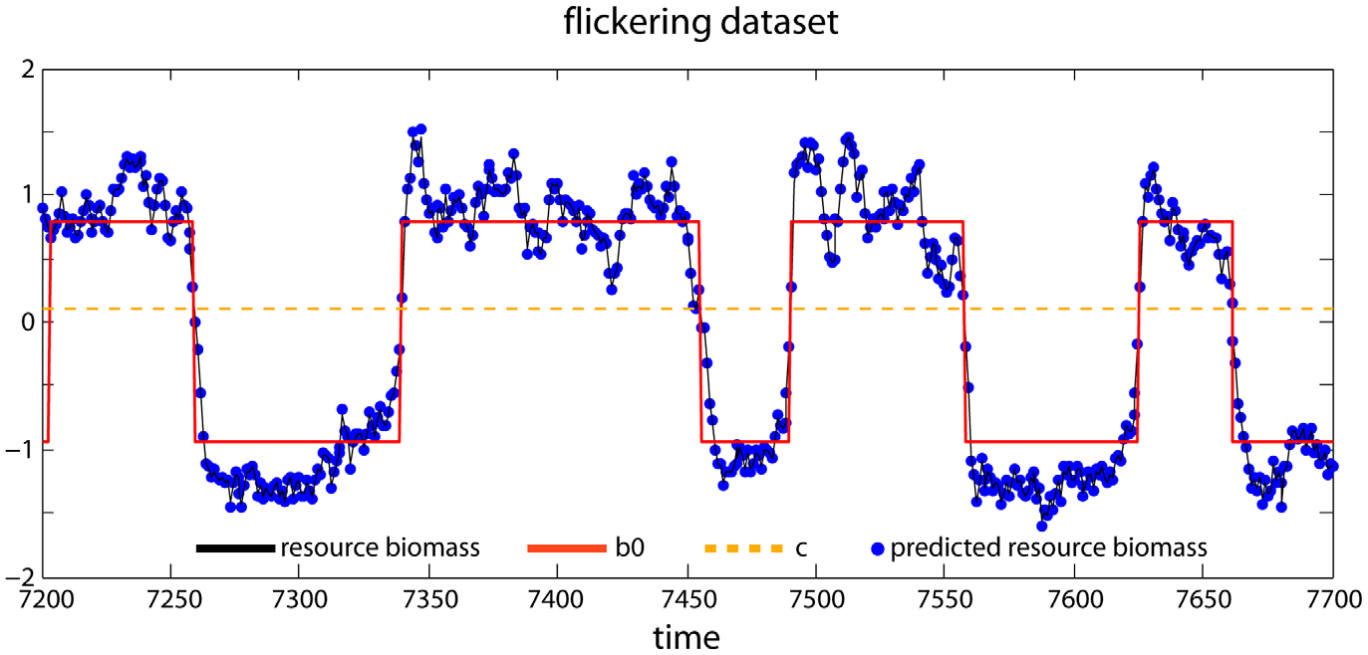
Fitting a threshold AR(3) model to the flickering dataset. Differences between the fitted trajectory (blue line) and the simulated data (black dots) are attributed to measurement error. The green line gives the estimates of *b*_0_(*t*) and *b*_0_'(*t*), and the yellow line gives the threshold *c* which separates the two AR(3) processes. Parameter estimates are: *b*_0_ = −0.941, *b*_0_' = 0.797, *b*_1_ = 1.192, *b*_1_' = 1.22, *b*_2_ = 0.069, *b*_2_' = −0.231, *b*_3_ = −0.326, *b*_3_' = −0.135, *c* = 0.1, *σ_ε_* = 0.125, and *σ_ε_* = 0.054, and the log likelihood = 238.954.

#### Skewness and Kurtosis

In some cases disturbances push the state of the system towards values that are close to the boundary between the two alternative states. Because the dynamics at the boundary become slow [6], we may observe a rise in the *skewness* of a time series- the distribution of the values in the time series will become asymmetric [29]. Just like variance, skewness can also increase because of flickering [6]. Skewness is the standardized third moment around the mean of a distribution and it is given by 
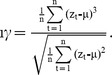
 Note that skewness may increase, or decrease, depending on whether the transition is towards an alternative state that is larger or smaller than the present state.

**Figure 9:**
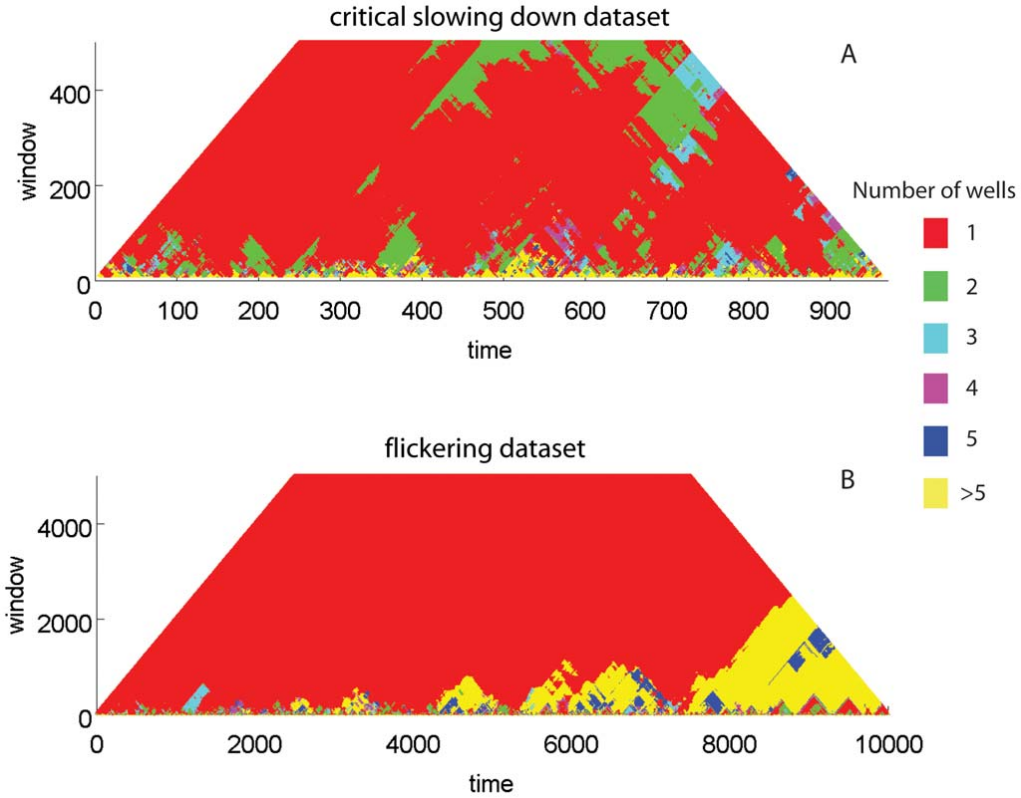
Potential analysis for the critical slowing down and flickering datasets. The potential contour plot represents the number of detected wells (states) of the system potential (x-axis corresponds to the time scale of the series, and y-axis is the size of the rolling window for detection). A change in the color of the potential plot along all time scales (vertically) denotes a critical transition in the time series.

Flickering or strong perturbations also make it more likely that the state of a system may reach more extreme values close to a transition. Such effects can lead to a rise in the *kurtosis* of a time series prior to the transition [25]; the distribution may become ‘leptokurtic’: the tails of the time series distribution become fatter due to the increased presence of rare values in the time series. Kurtosis is the standardized fourth moment around the mean of a distribution estimated as: 
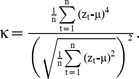


#### Conditional heteroskedasticity

Another measure of change in the pattern of variability in a time series is *conditional heteroskedasticity*. Conditional heteroskedasticity means that variance at one time step has a positive relationship with variance at one or more previous time steps. This implies that periods of high variability will tend to follow periods of high variability and periods of low variability will tend to follow periods of low variability [30], [31]. As variability tends to increase prior to a transition, conditional heteroskedasticity can serve as a leading indicator because the portion of a time series near an impending shift will appear as a cluster of high variability while the portion of the time series away from the shift will appear as a cluster of low variability [32]. Conditional heteroskedasticity is based on a Langrange multiplier test [30], [31], which is calculated by first extracting the residuals of a fitted model to the time series. Usually an autoregressive model of selected order is selected according to a measure of relative goodness of fit (e.g. the Akaike Information Criterion); then the residuals are squared, and finally the residuals are regressed on themselves lagged by one time step. A positive slope of the linear regression of the lagged residuals suggests conditional heteroskedasticity. The coefficient of determination of the regression *r^2^* is compared with a *χ^2^* distribution of one degree of freedom to assign the significance for the *r^2^*. The *χ^2^* value can be divided by the sample size to make it directly comparable to the *r^2^* value.

**Figure 10:**
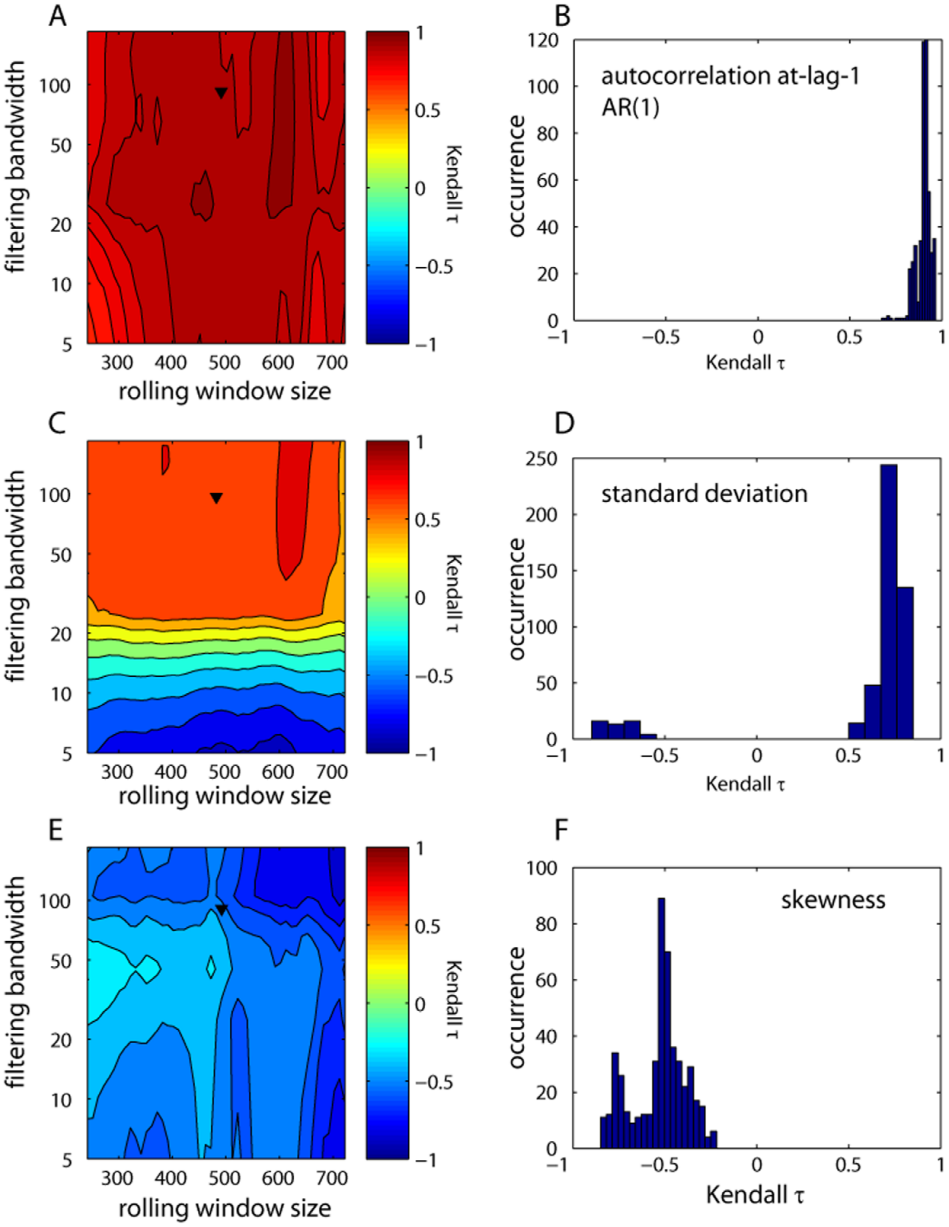
Sensitivity analysis for rolling window metrics (autocorrelation (AR1), standard deviation, and skewness) for the critical slowing down dataset. Contour plots show the effect of the width of the rolling window and Gaussian filtering on the observed trend in the metrics as measured by the Kendall’s τ (A, C, E). Upside triangles indicate the parameter choice used in the analyses presented in the text. The histograms give the frequency distribution of the trend statistic (B, D, F).

#### BDS test

The BDS test (after the initials of W. A. Brock, W. Dechert and J. Scheinkman) detects nonlinear serial dependence in time series [33].The BDS test was not developed as a leading indicator, but it can help to avoid false detections of critical transitions due to model misspecification. After detrending (or first-differencing) to remove linear structure from the time series by fitting any linear model (e.g. ARMA(p,q), ARCH(q) or GARCH(p,q) models), the BDS tests the null hypothesis that the remaining residuals are independent and identically distributed (i.i.d.) [10]. Rejection of the i.i.d. hypothesis implies that there is remaining structure in the time series, which could include a hidden nonlinearity, hidden nonstationarity or other type of structure missed by detrending or model fitting. As critical transitions are considered to be triggered by strong nonlinear responses, the BDS test is expected to reject the i.i.d. hypothesis in the residual time series from a system that is approaching a critical transition. The BDS test can be helpful as an *ad-hoc* diagnostic test to detect nonlinearities in time series prior to transitions: if the BDS test rejects the i.i.d. hypothesis and there is another strong leading indicator, then the detected early warning is less likely to be a false positive.

**Figure 11:**
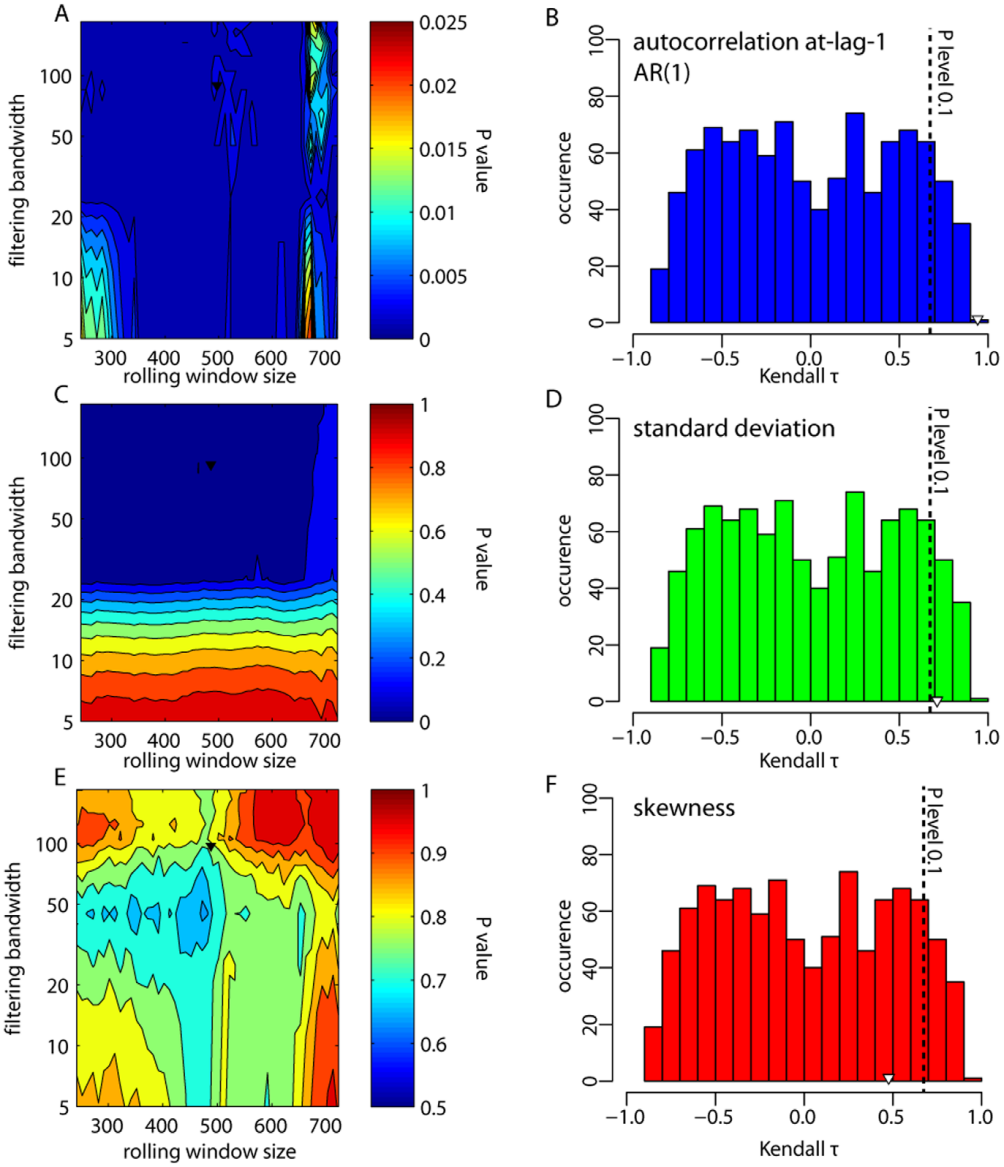
Significance testing for rolling window metrics (autocorrelation at-lag-1 (AR1), standard deviation, and skewness) for the critical slowing down dataset. (A, C, E) Contour plots of *P* values estimated from distributions of Kendall trend statistics derived from surrogate datasets for different rolling window lengths and sizes of Gaussian filtering. The surrogate datasets were produced from the best-fit ARMA model on the residual records of the critical slowing down dataset. *P* values were derived from probability distributions of the estimated trend statistic for a set of 1,000 surrogate datasets for a combination of a rolling window size and Gaussian filtering. For example, panels B, D, F show the distribution of Kendall trends estimated on 1,000 surrogates of the original residual dataset for rolling window size and Gaussian filtering as the one presented in the text. Black vertical lines indicate the *P* = 0.1 significance level and the upside open triangle is the actual Kendall trend estimated on the original residual dataset for rolling window size and Gaussian filtering as the one presented in the text (upside solid triangle in A, C, E).

### Model-based Indicators

#### Nonparametric drift-diffusion-jump models (DDJ models)

Often we do not know the underlying processes that generate the time series that we are analyzing for early warnings. Nonparametric drift-diffusion-jump models address this problem by fitting a general model that can approximate a wide range of nonlinear processes without the need to specify an explicit equation. Drift measures the local rate of change. Diffusion measures relatively small shocks that occur at each time step. Jumps are large intermittent shocks. Total variance combines the contributions of diffusion and jumps.

**Figure 12:**
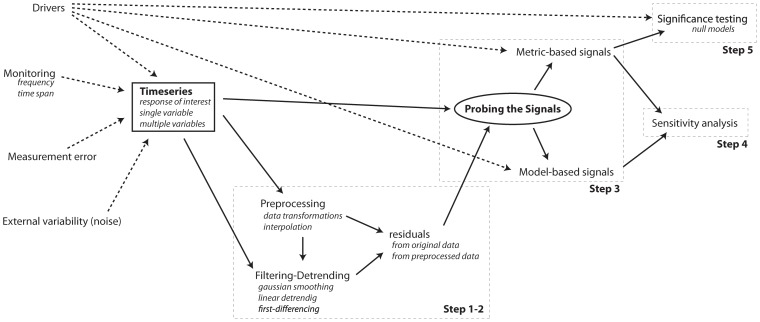
Flowchart for detecting early warning signals for critical transitions in time series. Solid arrows represent the procedure presented in the text. Dotted arrows represent interactions that affect different steps in the detection of early warning and that need to be taken into account in the interpretation of the signals.

The approach is to estimate terms of a drift-diffusion-jump model as a surrogate for the unknown data generating process [16]:
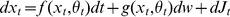
(2)


Here *x* is the state variable, *f*(·) and *g*(·) are nonlinear functions, d*W* is white noise, and *J* is a jump process. Jumps are large, one-step, positive or negative shocks that are uncorrelated in time. Equation 2 is assumed to be subject to a critical transition at a critical parameter value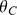, just as in equation 1. We assume that *x_t_* can be observed at discrete intervals of time Δ*t* that can be short, i.e. very high-frequency observations are possible.

**Table 3:** Rules of thumb for avoiding bottlenecks in detecting early warning signals in time series.

Method/Indicator	Preprocessing		Filtering/Detrending	Sensitivity		Significance
	*interpolation*	*transformations*		*rolling window size*	*filtering*	
*Autocorrelation at-lag-1*	necessary^1^	depends on data	+	+	+	null model^3^
*Detrended Fluctuation Analysis*	necessary^1^	no	+	+	−5	null model^3^
*Standard deviation*	no	depends on data	+	+	+	null model^3^
*Skewness*	no	depends on data	+	+	+	null model^3^
*Kurtosis*	no	depends on data	+	+	+	null model^4^
*Conditional Heteroskedasticity*	no	depends on data	−	+	−	built-in
*BDS test*	no	no	+	−2	+	bootstrapping
*Time-varying AR(p) models*	no	log-transform	+	−	−	built-in
*Nonparametric Drift-Diffusion-Jump models*	no	log-transform	−	−	−4	MC error estimates
*Threshold AR(p) models*	no	log-transform	−	−	−	built-in
*Potential Analysis*	no	no	n/a	+	−	null model^3^

1 only when there are too many missing values.

2 to be applied only for rolling windows of >500 points.

3 choice of null model contingent on system.

4 depends on bandwidth of Gaussian kernel smoother.

5 only polynomial detrending within rolling window.

[+: sensitive to; −: insensitive to; n/a: not applicable].

The data-generating process (eq 2) is unknown in the sense that the expressions for *f*(·) and *g*(·) are not known, *θ_t_* is neither known nor measured, the critical value *θ_c_* where *x* undergoes a catastrophic change is not known, and the parameters of the jump process are not known. From the time series, however, we can estimate drift, diffusion and jump statistics that may serve as leading indicators of the transition. We do this by assuming that high-frequency observations of the system in equation 2 can be approximated by fitting the drift-diffusion-jump model
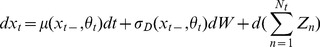
(3)


In this fitted model (eq. 3), the drift, diffusion, and jump functions track the slow and unknown changes in *θ_t_*. The drift function 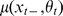
measures the instantaneous deterministic change in the time series. The diffusion function 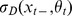 measures the standard deviation of relatively small shocks that occur at each time step. Jumps, the last term of equation 3, are relatively large shocks that occur intermittently. Jumps are characterized by an average magnitude 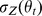 (where *Ζ_n_*∼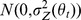) and the probability of a jump arriving in a small time increment *dt* is *λ*(*x_t_*, *θ_t_*)*dt*. The subscript *t-* in *μ*(·) and *σ_D_*(·) indicates that these functions are evaluated just before the time step. In practice, the drift, diffusion, and jump functions are estimated using nonparametric regression [34], [35]. The regression yields estimates of drift 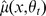, total variance 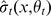, jump intensity 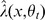, and the diffusion variance is given by 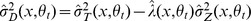, where 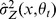 is the jump-variance function. In addition, we can estimate the conditional variance of *x* using standard nonparametric regression techniques. This conditional variance rises to infinity at a critical point caused by bifurcation in *f*(·), *g*(·) or both. The conditional variance function, 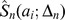, can be estimated as the difference between the second conditional moment and the square of the first conditional moment as 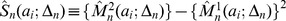 [16], [36]. An interesting feature of the drift-diffusion-jump model is that conditional variance and diffusion estimates may be useful for distinguishing bifurcations that occur in the drift from bifurcations that occur in the diffusion (so-called noise-induced transitions: an abrupt shift in the shape of the stationary distribution as in [37]). A bifurcation in the drift only may be indicated in advance by conditional variance but not diffusion. A bifurcation in the diffusion may be indicated by increases in both conditional variance and diffusion.

#### Time-varying AR(*p*) models

Time-varying autoregressive models provide a model-based approach for estimating time-dependent return rates in time series [38], which as we noted in the earlier section can act as an early warning of a critical transition. In time-invariant AR(*p*) models, the inverse of the characteristic root, *λ*, of a fitted AR(*p*) model [39] is similar in magnitude to the dominant eigenvalue of the Jacobian matrix computed at a stationary point of a deterministic discrete-time model [18]. Values of *λ* near zero imply that the state variable returns rapidly towards the mean; this central tendency diminishes as values approach one [22].

Time-varying AR(*p*) models assume that the coefficients of the AR(*p*) model can change through time, thereby allowing estimation of the time-dependent characteristic root as it varies along a time series up to a transition [38]. The general form of time-varying AR(*p*) models is
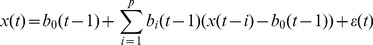
(4a)

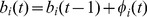
(4b)


Equation 4a is a standard AR(*p*) model with coefficient *b*_0_ determining the mean of the time series, autoregressive coefficients *b*_i_ determining the dynamics around the mean, and *ε*(*t*) giving the environmental variability associated with changes in the state variable; *ε*(*t*) is assumed to be a Gaussian random variable with mean zero and variance *σ* ^2^_ε_. Equation 4b allows the coefficients *b*_i_ to vary as random walks, with rates dictated by the variances *σ* ^2^ *_i_* of *φ_i_*(*t*).

We incorporate measurement error using the measurement equation
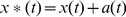
(5)in which x*(*t*) is the observed value of the state variable, *x*(*t*) is the “true” modeled value, and *α*(*t*) is a Gaussian random variable with mean zero and variance *σ* ^2^_α._ This makes it possible to factor out measurement error that could potentially obscure underlying dynamical patterns [38].

Together, equations 4a and 4b are a state-space model that can be fit using a Kalman filter [40]. Although we present the model assuming that data are sampled at equidistant points through time, the state-space structure allows for missing points. Fitting with a Kalman filter gives maximum likelihood parameter estimates, and likelihood ratio tests (LRT) can be used for statistical inference about the parameter estimates. Likelihood-based model selection such as Akaike’s Information Criterion (AIC) can also be used [38]. Because the variance components of the model, *σ* ^2^ *_i_*, are constrained to be zero, a standard LRT is overly conservative; the calculated *P*-values are too large, leading to acceptance of the null hypothesis that *σ* ^2^ *_i_* = 0 even when it is false. To correct for this, the LRT can be performed using the relationship that the twice the difference in log likelihoods between models differing by *q* in the number of terms *σ* ^2^ *_i_* they contain is given asymptotically by a 50∶50 mixture distribution of *χ* ^2^_(*q-*1)_ and *χ* ^2^ *_q_.* [41], [42]. Therefore, the corrected *P*-value is the average of *P*-values calculated from the two *χ* ^2^ distributions. Since *P*(*χ* ^2^_(*q-*1)_<*x*) is less than *P*(*χ* ^2^ *_q_*<*x*), this always leads to lower *P*-values than would be obtained from a standard LRT based on χ^2^ *_q_* alone.

#### Threshold AR(*p*) models

As described above, flickering occurs when a time series repeatedly crosses the domains of attraction of two alternative states. Identifying flickering can serve as an early warning for a permanent shift to an alternative state [6]. The difficulty lies in robustly estimating that a time series is jumping among two (or more) distinct states. Threshold AR(*p*) models are designed to identify these occasional transitions [38]. These models assume there are two underlying processes governing the dynamics in a time series, with the possibility that the state variable switches between them when it crosses a threshold. The two processes are described by two AR(*p*) models
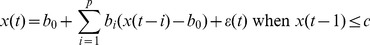
(6a)

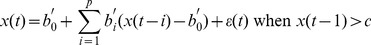
(6b)where *b_i_* and *b_i_*′ (*i* = 0, …, *p*) denote separate sets of coefficients. As with the time-varying AR(*p*) models (eqs 4), equation 5 is used to incorporate measurement error, and the Kalman filter is used to compute likelihoods in eqs 6, which in turn can be used for parameter estimation and model selection. In addition to the two sets of autoregression parameters *b*_i_ and *b_i_*′, parameters to be estimated are the threshold *c*, and the variance of the process error *σ* ^2^_ε_.

#### Potential analysis

An alternative way of probing the existence of alternative regimes in a time series is potential analysis. Just like threshold AR(p) models, this method in essence identifies flickering and serves as warning of the existence of alternative states. Potential analysis [43], [44] is a technique for deriving the shape of the underlying potential of a system. Potential analysis assumes that a time series may be approximated by a stochastic potential equation
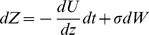
(7)where *dU/dz* is a polynomial potential of even order (2nd for one-well potential, 4th for double-well potential, etc.), *dW* is white noise of unit variance and intensity *σ*. The order of the best-fit polynomial in essence reflects the number of potential system states identified along the time series [43], [44].

Threshold AR(*p*) models and potential analysis are not, strictly speaking, early warnings for critical transitions, as flickering implies that the system already has undergone repeated state changes. Nonetheless flickering detection methods can robustly indicate the presence of alternative regimes during the period that the system has not permanently shifted to the alternative attractor.

### Datasets

We applied all methods to simulated time series - in which we are certain that a critical transition was crossed - rather than on real-world time series to illustrate the application of the methods across identical datasets. There are few available real-world time series that exhibit transitions, and for most of them there is no clear evidence that the transition is of the critical type we are treating here. Thus, for the illustrative purposes of our methodological paper, simulated datasets allowed us to compare the methods independently of uncertainties in the presence of a critical transition, data limitations, or insufficient data resolution that are common in empirical time series.

The two time series used were generated by a well-studied ecological model that describes the shift of a harvested resource to overexploitation [45], [46]. In the model, resource biomass *x* grows logistically and is harvested according to
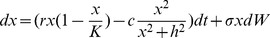
(8)where *r* is the growth rate, *K* is the population’s carrying-capacity, *h* is the half-saturation constant, *c* is the grazing rate and *dW* is a white noise process with intensity *(σx)^2^/dt*. In the deterministic case, when *c* reaches a certain threshold value (c ≈ 2.604), the ecosystem undergoes a critical transition to overexploitation through a fold bifurcation (Fig. 1A).

We simulated time series for two cases. In the first case (which we henceforth call the critical slowing down or CSD dataset), we increased grazing rate *c* linearly in 1,000 time steps from 1 to 2.6771 (just after the bifurcation). At approximately time step 970 the system shifted to overexploitation (Fig. 1B). Parameter values used were *r* = 1, *h* = 1, *K* = 10, *σ* = 0.03. The values were not parameterized for specific cases, but are similar to ones typically used in the literature (e.g. [45], [47], [48]). In the second case (which we henceforth call the flickering dataset), we again increased grazing rate *c* linearly from 1 to 2.6771 but in 10,000 time steps (Fig. 1C). In the flickering dataset, we additionally assumed a small time-correlated inflow *i* of resource biomass that was generated by a simple equation for red noise scaled to the resource biomass *x* [49]: 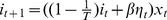, where *T* is a parameter that represents the time scale over which noise becomes uncorrelated ( = 20), and *β* the standard deviation ( = 0.07) of the normally distributed error term *η_t_*. Parameter values used were *r* = 1, *h* = 1, *K* = 10, *σ* = 0.15. For both scenarios we also included measurement error in the derived time series *x_obs,t_*  =  *x_t_* + *σ_obserr_ ε_t_*, where *σ_obserr_* is the standard deviation of the normally distributed error term *ε_t_*. We used *σ_obserr_*  = 0.1 for both the CSD and flickering datasets.

All simulated time series were produced in MATLAB R2011a using the software package GRIND (freely available at http://www.aew.wur.nl/UK/GRIND/). The estimation of the leading indicators was performed in R v.2.12.0 (http://www.r-project.org/) using R package *earlywarnings* (that can be downloaded at http://earlywarnings.r-forge.r-project.org/), except for the DFA and potential analysis, which were performed in MATLAB R2011a using Fortran and C computational kernels with shell scripts (can be obtained from VNL V.Livina@uea.ac.uk), and the time-varying AR(*p*) and threshold AR(*p*) models that were performed in MATLAB R2011a (and are available as supporting information in [38]). Further worked out examples can be also found at http://www.early-warning-signals.org.

## Results

We present results here assuming that the only available information to a practitioner is a time series derived from a system, which may be approaching a critical transition. The analysis is presented as a step-by-step procedure that starts with the preparation of the simulated time series (step 1, 2), the estimation of the leading indicators (step 3), and the testing of their sensitivity (step 4) and significance (step 5).

### Step 1 Preprocessing

To sensibly apply leading indicators, we first selected the part of the time series that preceded the potential transition. For most methods the estimation of the indicators takes place within rolling windows of predetermined size up to the end of the time series prior to the transition. We selected data up to time-step 970 in the CSD dataset (Fig. 1B). We used the whole time series of the ‘flickering dataset, as it was difficult to clearly identify when the transition took place. We ensured that there were no missing values and that all data were equally spaced in time (i.e. a regular time series). Regular time series are especially important in the case of leading indicators such as autocorrelation that estimate memory in time series. Interpolation can solve issues of missing values and irregular time series, but it can also result in spurious correlations, and checking interpolated records against the original time series to ensure that the density of interpolated points is constant along the time series should be considered [8]. Alternatively, points can also be dropped to obtain a regular time series. However, all the methods we used in this paper can also be applied to irregular time series as well as regular ones.

Equally important is the frequency of observations, that is, the time interval between values in the time series. In many cases data are recorded at different frequencies from the ones needed for the methods we illustrate. In principle, one needs data that are sampled at intervals shorter than the characteristic time scales of the slowest return rate of the system, especially when measuring indicators of critical slowing down [19], [50]. Averaging within non-intersecting windows of a given length results in records of longer time scales that may match the underlying dynamics of interest in the studied system [19], [27]. Choosing the length of the window to aggregate, however, depends on a fairly deep understanding of the dynamics of the system. In addition, aggregation also may solve the issue of missing values, although at the cost of losing data. Here, we did not need to aggregate our datasets because both were sampled in time scales that represented the characteristic time scale of the system we simulated.

We also transformed data where necessary. For example, we log-transformed (using log(z+1)) and in some cases also standardized [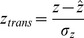] the flickering dataset, because of the presence of values close to zero or extreme values, respectively. We checked that data transformations did not change fundamentally the distribution of the original data, as it is exactly the deviations from constant normal distributions that the early warnings are sensitive to.

### Step 2 Filtering-detrending

Non-stationarities in the mean of the time series can cause spurious indications of impending transitions, especially for the metrics that are estimated within rolling windows. Additionally, time series may be characterized by strong seasonal periodicities, which, if not removed, impose a strong correlation structure on the time series. For all metrics that were estimated within rolling windows, we removed trends or filtered out high frequencies using Gaussian smoothing (autocorrelation, variance, skewness), simple linear detrending (DFA), or by fitting linear autoregressive models (conditional heteroskedasticity). When applying these or any other type of detrending or filtering (i.e. first-differences, removing running means, loess smoothing), care should be taken to not over-fit or filter out the slow dynamics (of interest) from the dataset [8]. Alternatively, one could also detrend within the rolling windows rather than the entire dataset. Lenton et al [27] have shown that results from the two approaches do not significantly differ.

### Step 3 Probing the Signals: Metric-based Indicators

#### Autocorrelation, variance and skewness

We estimated autocorrelation, variance (as standard deviation), and skewness within rolling windows half the size of the datasets (window size_CSD_ = 485 points, window size_flickering_ = 5,000 points) (Fig. 2). We did that after detrending the CSD dataset using Gaussian smoothing with bandwidth size 10% of the time series length (Fig. 2A). We used a sliding (overlapping) moving window based on the idea that indicators should be estimated as data are becoming available. Using nonoverlapping moving windows, however, would give similar results [27]. Autocorrelation at-lag-1 increased almost linearly up to the transition with a strong trend as estimated by Kendall’s τ (rank correlation) both for the original (τ = 0.911) and the residual (after detrending) datasets (τ = 0.944) (Fig. 2E). Standard deviations also increased in both original and detrended records as expected (Fig. 2G), while skewness generally decreased (τ = −0.436 for the original data, τ = −0.475 for the residuals after detrending), but in a somewhat irregular fashion (Fig. 2I). All indicators behaved according to our expectations for systems gradually approaching a critical transition, as may be seen in detail for all rolling window metrics associated to critical slowing down in Figures S1, S2 in the Supporting Information.

We estimated the same indicators for the flickering dataset on raw and log-transformed and standardized data (Fig. 2B, D). Autocorrelation (Fig. 2F) and skewness (Fig. 2J) increased, whereas standard deviation increased up to near time-step 8,000, after which it started to decline (Fig. 2H). In the flickering dataset, as the system was approaching the transition, excursions to the alternative attractor became more frequent (after time-step 2,000; Fig. 2B). The time series consisted of segments belonging to one or the other state (Fig. 1A). Autocorrelation was close to 1 and increased weakly (Fig. 2F). Progressively, segments belonging to the overexploited state became longer. As a result, standard deviation increased, but only up to the point where frequent transitions across the two attractors persisted (approx. up to time-step 8,000). After this point, the standard deviation decreased as only few points belonged to the underexploited state. Standardizing the data did not change the declining trend towards the end of the dataset, but only reduced its magnitude (Fig. 2H). The same few excursions to the underexploited state in the last part of the time series were responsible for the rise in skewness.

Autocorrelation at-lag-1 captured in a parsimonious way the changes in the correlation properties of a time series approaching a transition with respect to critical slowing down. A more complete picture of the changes in the spectral properties of the two datasets was also obtained by estimating the full variance spectrum using wavelet analysis (Fig. S3, S4 in the Supporting Information).

#### Detrended fluctuation analysis

The DFA indicator signaled an increase in the short-term memory for both datasets (Fig. 3B, D). It was estimated in rolling windows of half the size of the original record after removing a simple linear trend for both datasets. Despite oscillations, we could quantify its trend using Kendall’s τ. The values of the DFA indicator suggested that the CSD dataset was approaching the critical value of 1 (transition), whereas it was just below and above 1 in the flickering dataset (at the transition) implying that the latter system had exceeded the critical point and was nonstationary. These values resembled the approaching 1 (Fig. 2E) and close to 1 (Fig. 2F) values of autocorrelation at-lag-1.

#### Conditional heteroskedasticity

Conditional heteroskedasticity (CH) was estimated in rolling windows of 10% the size of the time series (Fig. 4). Within each rolling window we fit an autoregressive model selected using AIC from a suite of AR(p) models applied to the original data (Fig. 4A, B). Although measurement and process error remained constant in our datasets, we chose a relatively small rolling window size to minimize the chance of estimating an artificially large CH caused by increasing noise along the time series. We found significant CH (at *P* = 0.1) along the CSD dataset, which became consistently significant at the last part of the record (close to the transition) (Fig. 4C). In the flickering dataset, CH was always significant and its value even showed an increasing trend towards the end of the record (Fig. 4D).

#### BDS test

We removed the underlying linear structure by first-differencing, fitting an AR(1), or fitting a GARCH(0,1)) to the entire datasets after log-transforming. The remaining detrended data or the residuals were used to estimate the BDS statistic for embedding dimensions 2 and 3, and *ε* values 0.5, 0.75, and 1 times the observed standard deviation of the time series (Table 2). For each case, the significance of the BDS statistics was calculated using 1,000 bootstrap iterations. Results for both datasets showed significant BDS tests based on bootstrapping (Table 2). The only exception was the case of the residuals from the GARCH(0,1) model with embedding dimension 2 in the flickering dataset (Table 2). Thus, in general, the BDS statistic provided strong evidence for nonlinearity. In principle, we could have also applied the BDS statistic within rolling windows to flag a potentially increasing nonlinearity in a time series that is approaching a transition. However, when we tested this hypothesis, we did not get consistent results (not shown). The fact that the BDS test requires a large number of observations for a reliable estimate and that it is sensitive to data preprocessing and filtering choices are the main reasons that limit its use as a rolling window metric.

### Step 3 Probing the Signals: Model-based Indicators

#### Nonparametric drift-diffusion-jump models

The nonparametric DDJ model was not applied on rolling windows, but to the entire time series after log-transforming the data. We found an increase in conditional and total variance as well as in jump intensity in the CSD dataset (Fig. 5B, C, E) and a decrease in the diffusion term (Fig. 5D). The trends were noisy, but they became very clear when plotted against biomass values (due to smoothing) (Fig. 5F–I). For log-transformed values between 1.6 and 1.8, the indicators started to signal the upcoming transition. In the flickering dataset the indicators were very noisy and quite uninformative when plotted against time (Fig. 6B–E). However, after time-step 2,000, conditional variance, total variance, and jump intensity peaked and fluctuated between their maximum and minimum values. When we plotted the indicators versus biomass; the nonparametric variance related functions (Fig. 6F, G, I) increased as biomass declined from 2 to 0. These values corresponded roughly to the limit between the two alternative states (log biomass of zero and 2) (Fig. 6A). This example shows that plotting nonparametric indicators versus the monitored variable may be more informative than plotting indicators over time.

#### Time-varying AR(*p*) models

We fitted time-varying AR(*p*) models with *p* = 1, 2, and 3 to the CSD dataset after log-transforming and standardizing the data. For all cases, we computed time-varying AR(*p*) models for which only the mean, *b*_0_, was allowed to vary through time and compared them to AR(*p*) models for which both the mean and the autoregressive coefficients (*b*_i_, *i* ≥1) were allowed to vary with time. The log-likelihood ratio test (LRT) indicated that the models with varying autoregressive coefficients were significantly better than the mean-varying-only models (*χ* ^2^_0_+ *χ* ^2^_1_ = 37.1, *P*<0.0001 for AR(1); *χ* ^2^_1_+ *χ* ^2^_2_ = 44.3, *P*<0.0001, for AR(2); and *χ* ^2^_2_+ *χ* ^2^_3_ = 46.1, *P*<0.0001, for AR(3)). Comparing across models, the best fit was derived with the time-varying AR(1) model (ΔAIC = 2.2758 and 0.8059 for *p* = 2 and 3, respectively) (Fig. 7A); the difference in the AIC between the time-varying AR(1) and AR(3) models, however, was small (Fig. 7B). We therefore computed the inverse of the characteristic root *λ* of both time-varying AR(1) and AR(3) models at each point in the time series from the estimates of their autoregressive coefficients *b*_i_(*t*) (Fig. 7C, D). Values of *λ* approaching 1 imply critical slowing down, while values of *λ*>1 imply loss of stationarity. We found a clear increasing trend in *λ* (τ = 0.736) in the case of the time-varying AR(1) model (Fig. 7C), as the time series approached the transition. The trend in *λ* for the time-varying AR(3) model was weaker (τ = 0.164), less smooth, and in some cases exceeded 1, indicating strong excursions to nonstationarity (Fig. 6D). This suggests that the results of fitting time-varying AR(p) models might be more clear if simpler models (with lower *p*) are used.

#### Threshold AR(*p*) models

We fitted the threshold AR(*p*) model to only the flickering dataset as the method was developed to detect transitions in time series that jump between multiple states (Fig. 1B) [38]. The threshold AR(*p*) model was applied on log-transformed and standardized data. To simplify the analysis, we only used a subset of the original dataset, specifically observations between time step 7,200 and 7,700 (*n* = 500 points) (Fig. 8). We assumed that the time series was produced by two AR(*p*) processes of the same order. We tested orders of *p* = 1, 2, and 3 and found that the best-fitting model was an AR(3), with less-good fits for *p* = 1 (ΔAIC = 36.67) and *p* = 2 (ΔAIC = 1.75). The fit of the threshold AR(3) model was significantly better than the fit of a simple AR(3) (*χ* ^2^_4_+ *χ* ^2^_5_ = 27.79, *P*<0.0001). The tests of the same comparison were similarly significant for the AR(1) (*χ* ^2^_2_+ *χ* ^2^_3_ = 18.07, *P*<0.0004) and AR(2) (*χ* ^2^_3_+ *χ* ^2^_4_ = 20.88, *P*<0.0003) (Fig. 8). The consistent results from the fitted threshold AR(*p*) models confirmed that the dataset was characterized by two distinct states, which suggests that in the future the system may eventually stabilize in the alternative state.

#### Potential analysis

Contrary to the threshold AR(*p*) model fitting, potential analysis was performed within rolling windows of different size (ranging from 10 to half the size of the dataset). We applied it on untransformed data for both CSD and flickering datasets (Fig. 9). In the CSD dataset, we found that the method detected predominantly 1 state along the entire time series regardless of window size (red color Fig. 9A), but, interestingly, also identified two states especially for large size rolling windows (green color Fig. 9A). In the flickering dataset, one state was largely identified for most of the time series, except from the last 2,000 points onwards when multiple states where identified (Fig. 9B). Such high number of detected states meant that, in principle, the data were on the edge of having no clear potential.

### Step 4 Sensitivity Analysis

The utility of each of the leading indicators depends on the characteristics of the particular datasets we explored, and the specific choices made when performing the analyses, e.g., data transformations or detrending/filtering. Thus, it is necessary to check the robustness of our results to such choices. Here we did this for autocorrelation, standard deviation and skewness in the CSD dataset to illustrate that assumptions over specific parameters in the estimation of leading indicators need to be accompanied by a sensitivity analysis. In particular, we investigated the robustness of our rolling window metric results to the size of rolling windows and the degree of smoothing (filtering bandwidth). For this, we estimated autocorrelation, standard deviation and skewness in window sizes ranging from 25% to 75% of the time series length in increments of 10 points, and for bandwidths ranging from 5 to 200 in increments of 20 [8]. We quantified trends for all combinations of these two parameters using Kendall’s τ - although other quantifications of the trends can also be used. It is important to note that increasing but oscillating trends in the indicators can produce weak or even negative τ’s, and thus special care should be taken in the interpretation of the results of the sensitivity analysis.

We found that autocorrelation at-lag-1 increased rapidly regardless of the bandwidth choice and the size of the rolling window (Fig. 10A, B). We found similar strong trends for standard deviation, even if there were negative trends identified for small bandwidths (Fig. 10C, D). This was probably due to the fact that small bandwidths over-fit the data and removed most of the variability, which the standard deviation was expected to capture. Trends in skewness were weaker, but mostly as expected (Fig. 10E, F). Although such sensitivity plots can guide in selecting the bandwidth and rolling window size to maximize the estimated trend, the specific choices of these two parameters should always be done according to the characteristics of the time series used. For instance, the choice of the rolling window size depends on a trade-off between availability of data and reliability of the estimation of the indicators [8]. We also did a sensitivity analysis for DFA exponents for both datasets (Fig. 3 E, F). The DFA exponent showed strong positive trends for both datasets. Similar sensitivity analysis on specific choices of parameters used should be conducted for any leading indicator applied to any time series.

### Step 5 Significance Testing

Although sensitivity analysis was important for testing the robustness of our results, it was equally important to test the significance of our results. Significance testing is especially relevant for identifying false positives (or type I errors): that trends in the indicators are not due to random chance. Some of the methods have built-in significance testing procedures (like conditional heteroskedasticity and the BDS test). The model-based indicators also allow for formal significance testing and model selection (e.g., the time-varying and threshold AR(*p*) models, and the potential analysis). The nonparametric DDJ model can be simulated after fitting to produce pseudo-data in Monte Carlo simulations that can be refitted to compute error estimates for total variance and jump intensity from the ensemble of fits [16].

For the remainder of the rolling window metrics, there is no built-in way to test a null hypothesis. The problem lies in the difficulty of specifying the exact null hypothesis, as it is not clear which particular data generating process could be used as the null model. Here, we suggest that the simplest null hypothesis one could imagine is that the trend estimates of the indicators are due to chance alone. To test this null hypothesis, we produced surrogate datasets to compare trend estimates in the original record with trend estimates obtained from records that have the same correlation structure and probability distribution as the original dataset, but that were produced by linear stationary processes [8]. Surrogate datasets can be obtained by different approaches, including generating data with the same Fourier spectrum and amplitudes [8], [51], or generating data from the simplest fitted linear first-order autoregressive model. Although these are only some of the ways surrogate data can be produced to test for trends [52], we used here a more general approach. We fit the best linear autoregressive moving average model (ARMA(*p*,*q*)) based on AIC to residuals (after detrending/filtering), then generated 1,000 simulated datasets of the same length as the residual time series. For each simulated dataset, we estimated the trend of the rolling window metric (in particular we only tested for autocorrelation at-lag-1, standard deviation, and skewness) using Kendall’s τ. We compared the Kendall τ of the original data to the number of cases in which the statistic was equal to or smaller than the estimates of the simulated records, *P* (*τ* ^*^≤*τ*). We estimated this probability for all combinations of bandwidth and rolling window size as we did for the sensitivity analysis (Fig. 10).

We found that the increasing trends for autocorrelation at-lag-1 were significant (*P*<0.025) for any combination of rolling window size and filtering bandwidth (Fig. 11A, B), and *P*≤0.001 for the parameters we used in Fig. 1. Similar significant trends were estimated for the standard deviation with a few exceptions (Fig. 11C, D, *P* = 0.073 for original choice of parameters in the CSD dataset). Skewness trends were not significant, however (Fig. 11E, F, *P* = 0.8 for original choices of CSD dataset).

Whatever statistical testing is used, the conclusions will depend on the specific model chosen either to fit data in the case of model-based approaches, or to produce simulated records for metric-based approaches. Thus, when interpreting significance testing of leading indicators estimates, one needs to take these considerations into account.

## Discussion

In this paper we applied a range of proposed early warning signals for critical transitions to two simulated time series. We presented a framework of combining *metric-based* indicators and *model-based* indicators to time series data to successfully identify an upcoming critical transition (Fig. 12). We found that there was no single best indicator or method for identifying an upcoming transition in line with previous studies [16], [48], [53]. Also, all methods required specific data-treatment to yield sensible signals (Table 3). This observation across all methods for the same datasets stresses that a combination of approaches is the best way to determine whether there is a robust signal of an imminent transition in a time series.

We only analyzed time series of a simulated ecological variable (resource biomass), however, our methods can equally be applied for time series representing any other response of interest: biological (e.g. gene expression), climatic (e.g. daily temperature), physiological (e.g. respiratory rhythm), social (e.g. numbers of tweets), or financial (i.e. price of a stock). In all these cases, if the system in question undergoes a critical transition through a fold bifurcation, we expect the indicators to behave in a similar way as we presented here. It is worthwhile testing this expectation on simulated data from such disparate systems, or even testing the indicators for other types of critical transitions than the ones we treated here. The big challenge for the future, though, is to test the indicators on real-world time series. Most studies so far have treated only subsets of indicators on real time series. Using our framework to test indicators on real-world time series will highlight limitations in the application and interpretation of the indicators other than the ones we presented here. Future work is needed towards this direction.

Nonetheless, our framework of combining metric-based and model-based indicators to detect critical transitions is encouraging as it may reduce the chance of false alarms. For instance, a systematic increase in the external noise over the period leading up to a shift can signal an increase in variance indicators [29], but not memory indicators (Table 1). However, cross-validation does not exclude the possibility of ‘missed alarms’ - cases where the indicators will not signal an approaching transition. Missed alarms can occur especially for transitions between attractors induced by major perturbations, or chaotic dynamics far from local bifurcation points [15]. Importantly, early warnings can only signal an upcoming transition if conditions slowly move the system towards a bifurcation. This excludes their applicability for instance to situations in which external forcing changes are faster than the response rate of the system [14].

Clearly the possibility of false alarms or missed signals is difficult to eliminate. Even in the case of a simulated time series that is known to be approaching a transition, certain methods may not be very informative [48]. By using single realizations from model-generated time series, we have been able to compare different methods on typical dynamical behaviors that occur before a critical transition. It will be worthwhile to robustly evaluate the performance of the different methods to quantify their reliability in signaling upcoming transitions. This could be done either statistically, by estimating indicators on multiple realizations of model generated time series, or by blind-testing the different methods on multiple datasets (e.g. [54]). Our results caution, however, that in all cases the performance of any method, as well as the interpretations based on them, will strongly depend on the characteristics of the actual time series tested.

In view of the limited scope of generic early warning signals, specific knowledge of the system may be of great use to reduce uncertainty. For instance, information about the noise level can help correct early warning estimates [55], or information on measurement error can be incorporated in the time-varying and threshold AR(*p*) model-based methods to improve estimation [38]. However, the most important source of information is insight about the drivers (or slow variables) that affect the stability properties of the system. For example, incorporating dynamics of drivers in the general model structure of time-varying AR(*p*) or Drift-Diffusion-Jump nonparametric model-based methods can greatly improve the estimation of early warnings. In other cases, information on drivers may offer evidence in support of concordant indicators, or can help explain why different indicators give different results [5].

In addition, driver-response relationships can help build mechanistic models of how the system works. On the one hand, such models can be used for estimating early warnings directly. For instance, generalized models in the presence of limited data can help measure critical slowing down [56]. Early warnings combined with dynamic linear modeling also can improve the estimation of indicators when information on mechanisms is limited [28]. On the other hand, such models can be used for building null models to statistically test the significance of most indicators.

Unfortunately, knowledge to build such specific mechanistic models is limited in most cases. In the extreme case, the only source of information available is a time series of a response variable, as in the datasets we analyzed here. Of course, in practice there are typically some other available data on drivers, triggers, or other processes, but mechanistic understanding differs widely between systems. The families of metric- and model-based generic early warnings offer the opportunity to identify upcoming transitions even in the absence of any specific knowledge over the underlying generating process. Moreover, advances in data collection and high frequency monitoring can increase confidence in the potential of using early warnings in cases where mechanistic understanding is limited.

Such high frequency observations might also lead to considering alternative methods. For instance, for high frequency data with inherent periodicities, such as electroencephalogram (EEG) time series of neural activity, Fourier decomposition or wavelet analysis may prove useful. In the Supporting Information (Fig. S4), we illustrate the potential application of wavelet analysis for such data, but such period decomposition techniques have not yet been fully tested for detecting critical transitions.

In other cases, observations of multiple time series may be available. Monitoring >1 species in a community, or measuring the activity of numerous neural cells yields multivariate time series that could enhance our ability to detect approaching transitions. In such case, multivariate indices (like covariances) can be used [23], or extensions of the univariate time-varying AR(p) models to multivariate analogs have been proposed [38]. Similarly, spatial data can be of added value as spatial information may also provide early warning signals. Some of these signals are in fact mathematical analogs of the signals in time series indicators (spatial variance [57], spatial skewness [58], spatial autocorrelation [59]), while others can be system-specific, such as patch shape [60] and patch size distribution [61], [62]. These spatial indicators can be combined with the indicators for time series presented here to provide more reliable signals [53]. We treat spatial indicators in depth in a separate paper.

Clearly we face formidable uncertainty when it comes to making decisions in the prospect of potential upcoming transitions. This uncertainty stems from multiple factors including imprecise forecasts, insufficient data, and hidden nonlinearities [63], [64] as well as from the peculiarities in perception and tolerance of risk. Our framework for using early warning signals may help pave the way to a more robust evaluation of the risk of imminent transitions. Testing our framework in real world datasets is the next step towards that direction.

## Supporting Information

Figure S1 **Rolling Window Metrics: Autocorrelation at-lag-1 (ACF(1) and AR(1)), Spectral ratio, Return rate, Standard Deviation, Coefficient of Variation, Skewness, Kurtosis for the filtered critical slowing down dataset.**
(TIF)

Figure S2 **Rolling Window Metrics: Autocorrelation at-lag-1 (ACF(1) and AR(1)), Spectral ratio, Return rate, Standard Deviation, Coefficient of Variation, Skewness, Kurtosis for the unfiltered (original) critical slowing down dataset.**
(TIF)

Figure S3 **Spectral densities and spectral exponent for the critical slowing down and flickering datasets.**
(TIF)

Figure S4 **Wavelet analysis for the critical slowing down and flickering datasets.**
(TIF)
