# Supplementary figures and images for: Methods for Detecting Early Warnings of Critical Transitions in Time Series Illustrated Using Simulated Ecological Data
Source: PLoS One. 2012 Jul 17;7(7):e41010. doi: 10.1371/journal.pone.0041010 (PMC3398887; doi:10.1371/journal.pone.0041010)

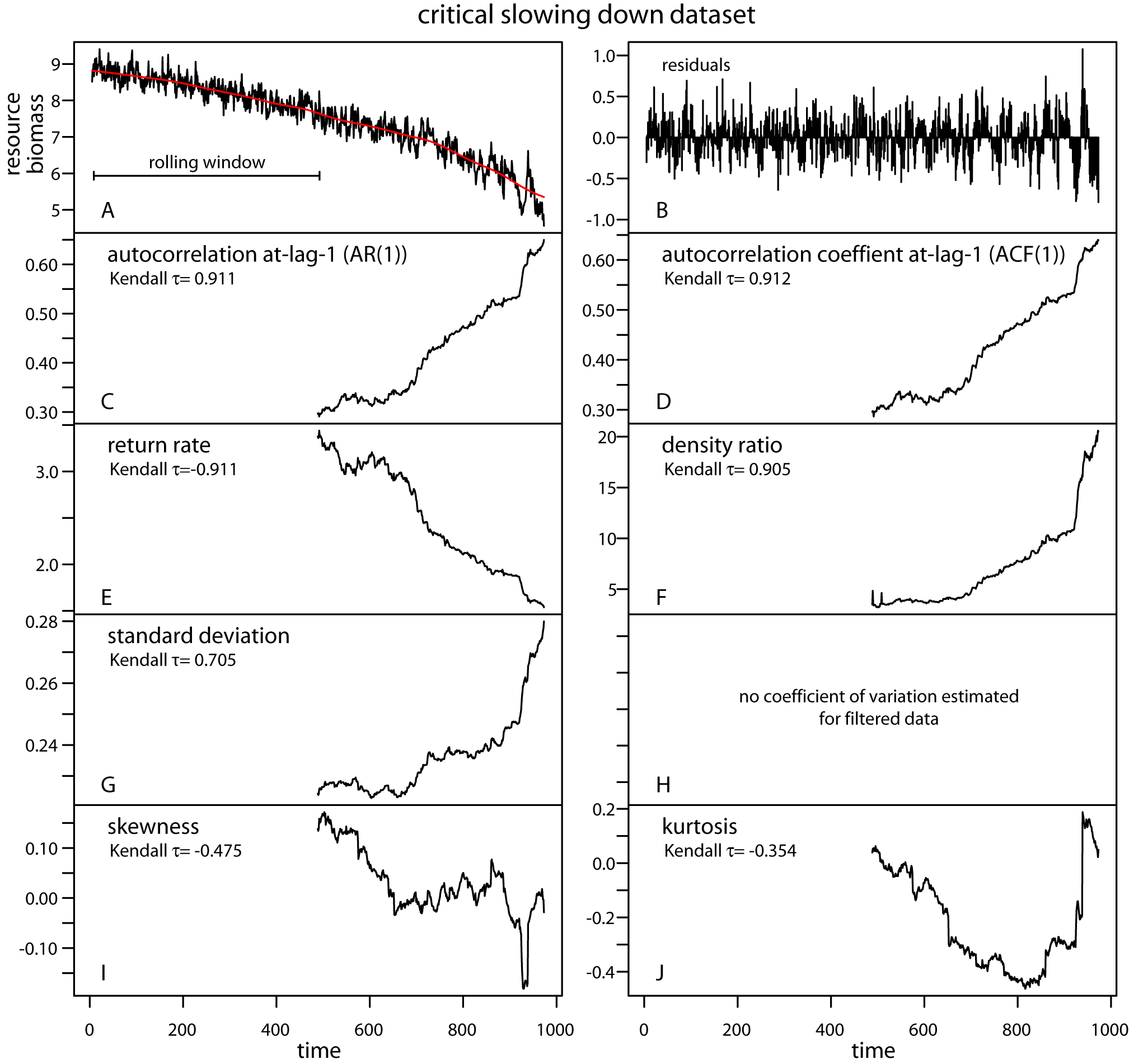

Supplement: Figure S1 — Rolling Window Metrics: Autocorrelation at-lag-1 (ACF(1) and AR(1)), Spectral ratio, Return rate, Standard Deviation, Coefficient of Variation, Skewness, Kurtosis for the filtered critical slowing down dataset. (TIF) [file pone.0041010.s001.tif]

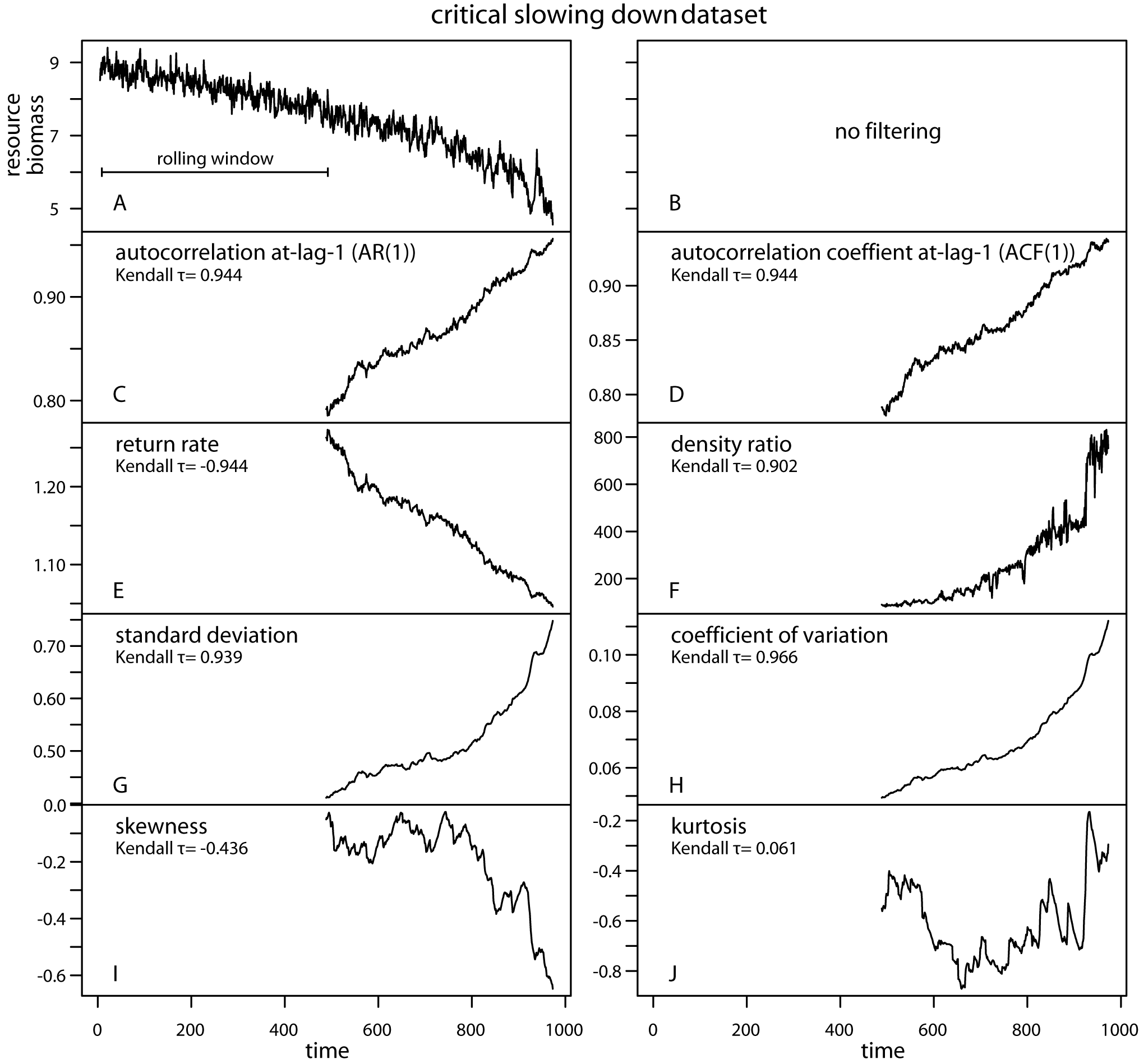

Supplement: Figure S2 — Rolling Window Metrics: Autocorrelation at-lag-1 (ACF(1) and AR(1)), Spectral ratio, Return rate, Standard Deviation, Coefficient of Variation, Skewness, Kurtosis for the unfiltered (original) critical slowing down dataset. (TIF) [file pone.0041010.s002.tif]

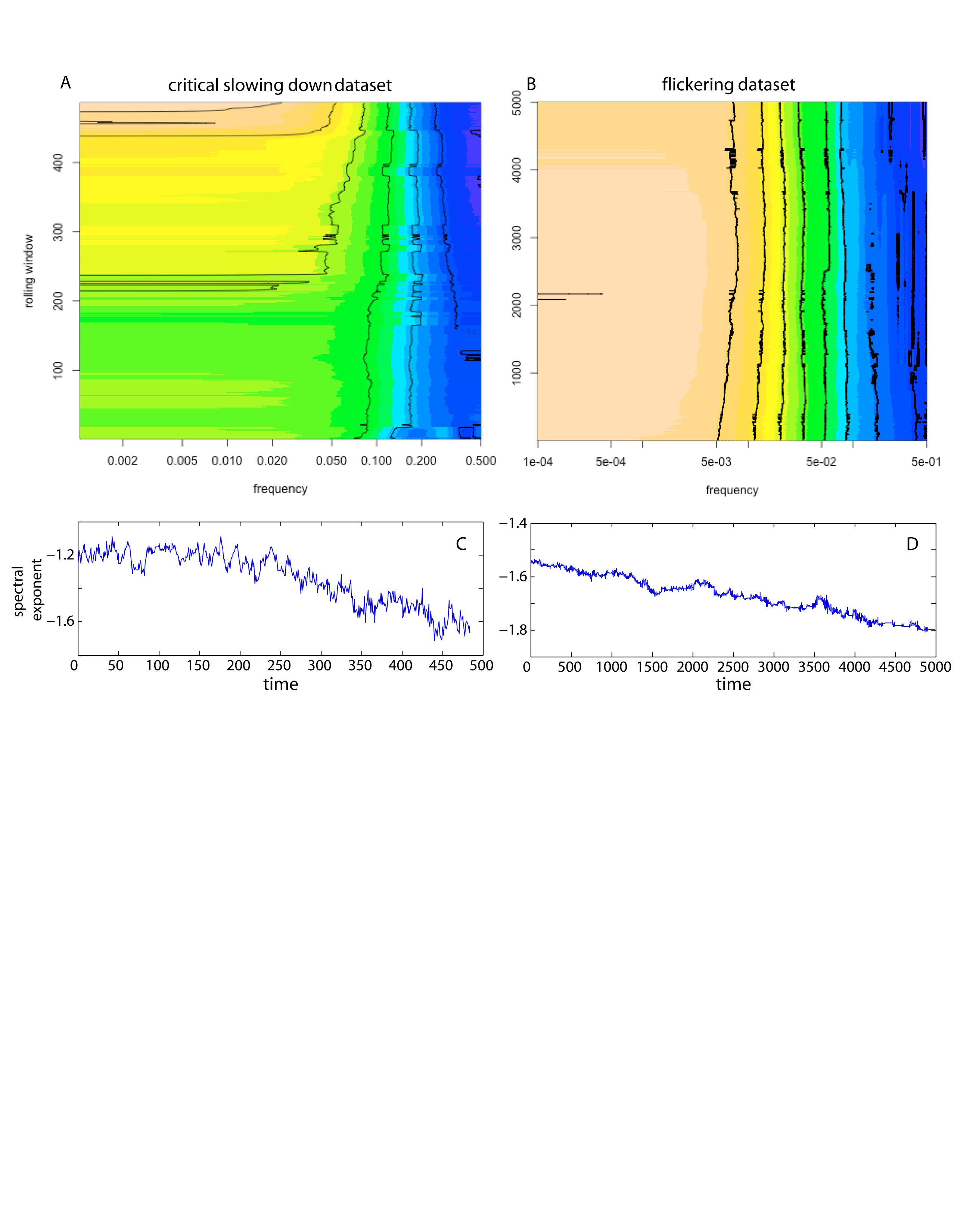

Supplement: Figure S3 — Spectral densities and spectral exponent for the critical slowing down and flickering datasets. (TIF) [file pone.0041010.s003.tif]

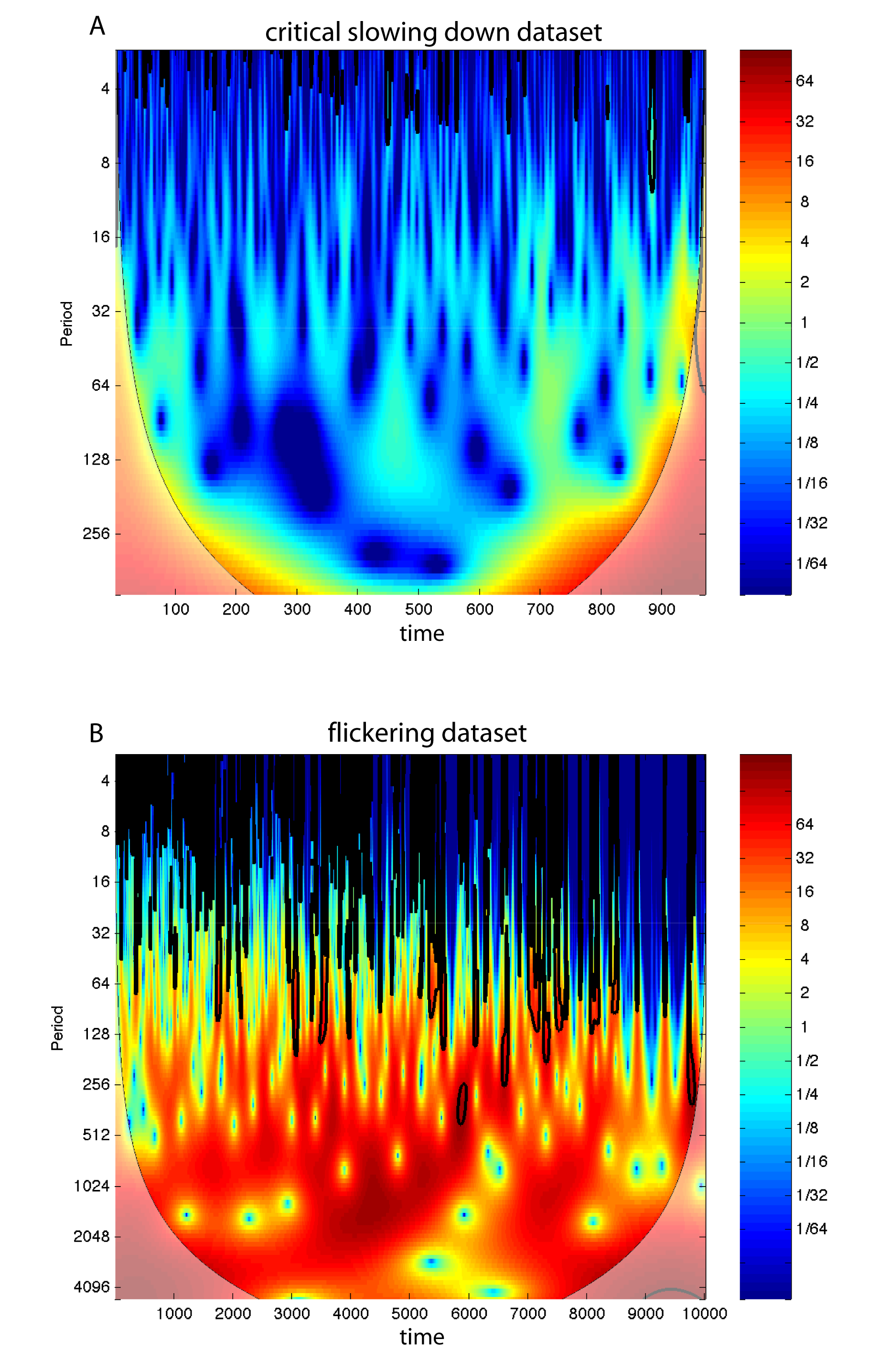

Supplement: Figure S4 — Wavelet analysis for the critical slowing down and flickering datasets. (TIF) [file pone.0041010.s004.tif]
